# Homeostatic Cytokines Drive Epigenetic Reprogramming of Activated T Cells into a “Naive-Memory” Phenotype

**DOI:** 10.1016/j.isci.2020.100989

**Published:** 2020-03-19

**Authors:** Guido Frumento, Kriti Verma, Wayne Croft, Andrea White, Jianmin Zuo, Zsuzsanna Nagy, Stephen Kissane, Graham Anderson, Paul Moss, Frederick E. Chen

**Affiliations:** 1 Institute of Immunology and Immunotherapy, University of Birmingham, Birmingham, UK; 2 NHS Blood and Transplant, Birmingham, UK; 3 Centre for Computational Biology, University of Birmingham, Birmingham, UK; 4 Institute of Inflammation and Ageing, University of Birmingham, Birmingham, UK; 5 Technology Hub, University of Birmingham, Birmingham, UK; 6 Centre for Clinical Haematology, University Hospitals Birmingham NHS Foundation Trust, Birmingham, UK; 7 Clinical Haematology, Barts Health NHS Trust, London, UK; 8 Blizard Institute, Queen Mary University London, London, UK

**Keywords:** Biological Sciences, Molecular Biology, Immunology

## Abstract

Primary stimulation of T cells is believed to trigger unidirectional differentiation from naive to effector and memory subsets. Here we demonstrate that IL-7 can drive the phenotypic reversion of recently differentiated human central and effector memory CD8^+^ T cells into a naive-like phenotype.

These “naive-revertant” cells display a phenotype similar to that of previously reported stem cell memory populations and undergo rapid differentiation and functional response following secondary challenge. The chromatin landscape of reverted cells undergoes substantial epigenetic reorganization with increased accessibility for cytokine-induced mediators such as STAT and closure of BATF-dependent sites that drive terminal differentiation. Phenotypic reversion may at least partly explain the generation of “stem cell memory” CD8^+^ T cells and reveals cells within the phenotypically naive CD8^+^ T cell pool that are epigenetically primed for secondary stimulation. This information provides insight into mechanisms that support maintenance of T cell memory and may guide therapeutic manipulation of T cell differentiation.

## Highlights

• γ-chain cytokines revert newly differentiated CD8^+^ T cells to a naive-like phenotype
• These “naive-revertant” are primed for secondary challenge
• Their chromatin landscape is reminiscent of memory cells
• Specific signaling pathways and transcription factors are involved

## Graphical Abstract

**Figure:**
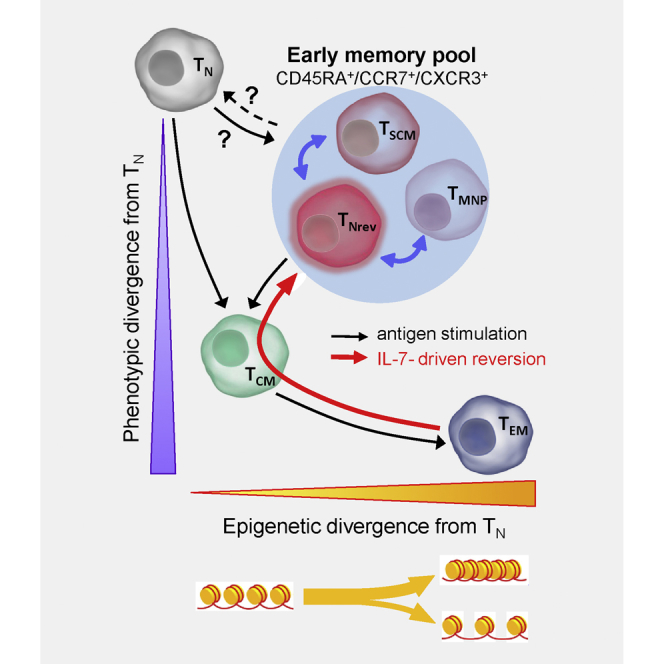

## Introduction

The development of T cell memory is essential for long-term health but it remains uncertain how this population is maintained over the many decades of human lifespan. Current models of CD8^+^ T cell differentiation propose a unidirectional and irreversible pathway whereby initial antigen stimulation triggers sequential differentiation of naive (T_N_) cells into central memory (T_CM_), effector memory (T_EM_), and effector (T_Eff_) T cells (Klebanoff et al., 2006). T cells gradually acquire increasing effector function but this is associated with a progressive reduction in the capacity for differentiation and self-renewal, i.e., “stemness.” In settings of persistent antigenic stimulations T cells may progressively lose effector functions and proliferative capacity such that they eventually become exhausted.

Two subsets of phenotypically naive CD8^+^ T cells with features of memory cells have recently been described. “T-memory stem cells” (T_SCM_), which display enhanced capacity for self-renewal and multipotent proliferative potential (Gattinoni et al., 2011), are believed to be minimally differentiated and located between T_N_ and T_CM_ in the differentiation pathway (Gattinoni et al., 2017). “Memory T cells with naive phenotype” (T_MNP_) also exhibit broad polyfunctional capability (Pulko et al., 2016) and are thought to be functionally imprinted at an early stage of differentiation between CD8^+^ T_N_ and T_CM_ subsets. Despite sharing many characteristics, T_SCM_ and T_MNP_ differ in their extended phenotype and it is uncertain if they represent distinct and stable subsets or derive from a common precursor with phenotypic plasticity. Although CD8^+^ T_SCM_ can be produced *in vitro* by activating T_N_ cells in the presence of interleukin (IL)-7, IL-21, and the glycogen synthase-3β inhibitor TWS119 9 (Sabatino et al., 2016), the physiological mechanisms leading to the generation of both these cells and T_MNP_ are largely unknown.

Given the importance of cytokines as key regulators of T cell-mediated immunity, we analyzed the effect of different cytokines on T cell differentiation after primary stimulation, using T cells from human cord blood (CB), which are unlikely to have encountered antigen and therefore have a very low frequency of T_SCM_ (Gattinoni et al., 2011). We observed that recently differentiated CD8^+^ memory T cells can undergo lineage reversion to a naive-like phenotype when exposed to γ-chain cytokines and that these naive-revertant cells share extensive phenotypic and functional characteristics with both T_SCM_ and T_MNP_. This work describes a new pathway of T cell differentiation and provides a unifying theory for the generation of T cells with a “naive-memory” profile.

## Results

### IL-7 Induces Recently Differentiated CD8^+^ Memory T Cells to Revert to a Naive-like Phenotype

**Figure 1:**
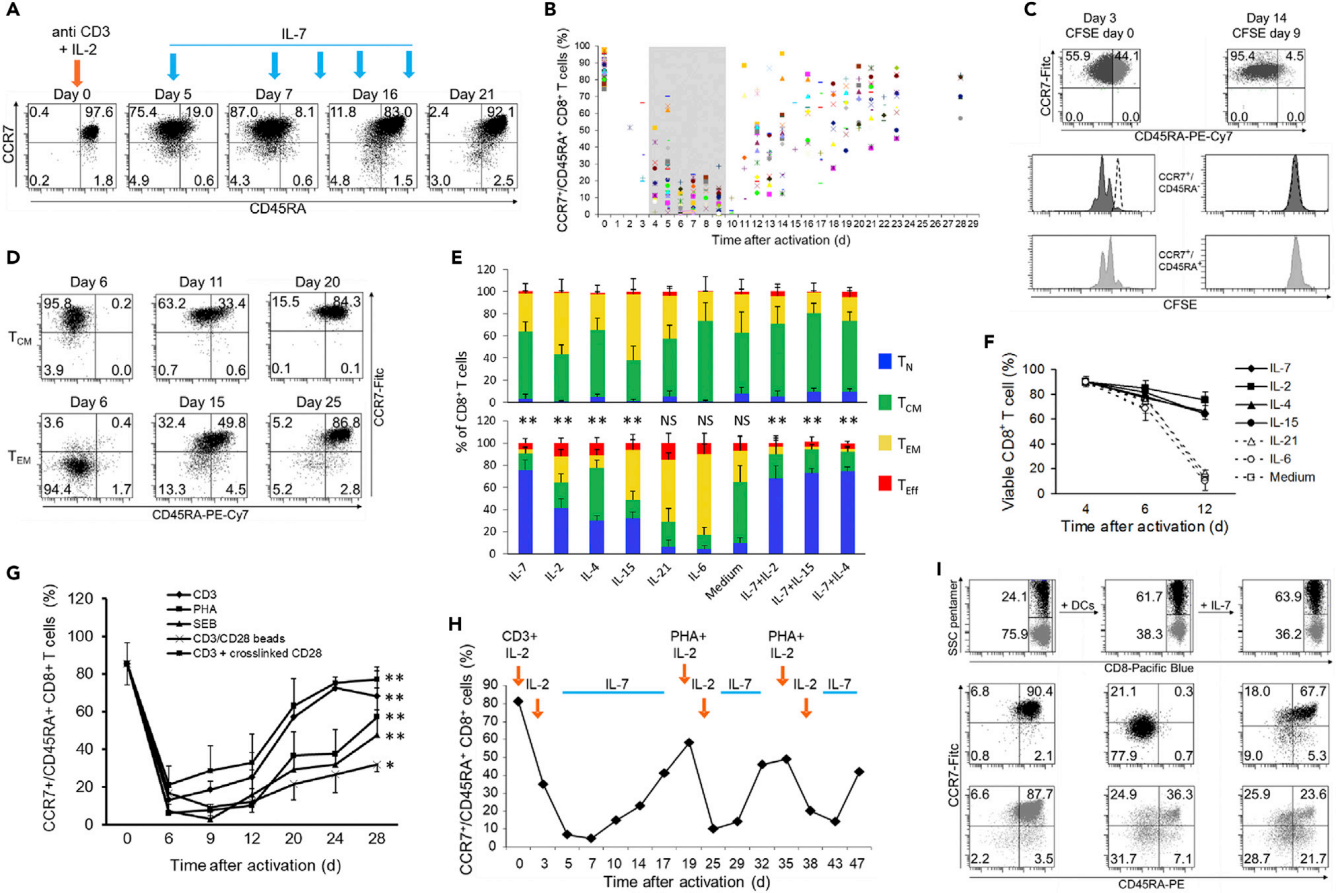
IL-7 Induces Reversion of Recently Differentiated Memory CD8^+^ T Cells to a Naive-like Phenotype (A) Flow cytometric analysis of phenotypic changes in CD8^+^ T cells after activation and successive incubation with 25 ng/mL IL-7. CBMCs were activated with anti-CD3 plus IL-2, and when the percentage of CD8^+^ T_N_ dropped below 20%, in this case day 5, cultures were maintained in IL-7. Numbers indicate the percentage of cells in each quadrant. Single representative experiment out of 50. (B) Kinetics of phenotype reversion of CD8^+^ T cells from the 50 different CB samples. Each symbol represents one sample. The shaded area indicates the interval of time when IL-7 was added for the first time. (C) CD8^+^ T cell proliferation after activation and IL-7 administration. CBMCs were stained with CFSE either before activation (left panels) or at day 9, during phenotype reversion (right panels). At the indicated time points, cell phenotype and CFSE content were assessed for T_N_ (light gray dots) and T_CM_ (dark gray dots). Dashed lines indicate basal content in CFSE. Single representative experiment out of three. (D) Flow cytometry evaluation of IL-7-dependent phenotype reversion in recently differentiated T_CM_ and T_EM_. After activation T_CM_ and T_EM_ were negatively selected. The two cell subpopulations were then incubated with IL-7 and monitored for phenotype changes over time. Single representative experiment out of three, for each subset. (E) The effect of different cytokines on phenotype reversion. CBMCs were activated, and when the percentage of CD8^+^ T_N_ dropped below 20% the indicated cytokines were added. The percentage of the cells in the different subsets is shown when the percentage of CD8^+^ T_N_ reached the nadir (upper panel) and afterward, when it reached the plateau (lower panel). Data from six samples. Paired t test analysis between the T_N_ levels at nadir and plateau. ∗∗ = p < 0.001. (F) Viability of cells incubated with different cytokines. Activated CBMCs were incubated from day 4 with each cytokine or medium, and CD8^+^ T cell viability was evaluated by flow cytometry using 7-AAD uptake. Data are represented as means ± 1SD of three samples. (G) The kinetics of phenotype reversion of CD8^+^ T cells activated with different artificial stimuli. Data are represented as means ± 1SD of three samples. Paired t test analysis between the T_N_ levels at nadir and plateau. ∗ = p < 0.05, ∗∗ = p < 0.001. (H) The kinetics of phenotype reversion of CD8^+^ T cells undergoing successive cycles of activation/IL-7 incubation. Newly generated CD8^+^ T_Nrev_ cells were twice re-stimulated with PHA and induced to revert twice with IL-7 when the percentage of T_N_ dropped below 20%. Single representative experiment out of three. (I) Flow cytometry analysis of phenotype changes of CD8^+^ T_Nrev_ upon activation with the cognate antigen. CB T lymphocytes were activated, retrovirally transduced with the SSC-TCR, and induced to revert their phenotype with IL-7 (left panels). Afterward, cells were incubated with peptide-pulsed DCs (central panels). IL-7 was then added again, driving the transduced cells to revert their phenotype (right panels). Plots were gated on CD8^+^ T cells. The upper panels show the percentage of transduced (black dots) and non-transduced (gray dots) CD8^+^ T cells.

CB mononuclear cells (CBMCs) were activated with anti-CD3 plus IL-2, and the differentiation stage of CD8^+^ T cells was evaluated by CD45RA and CCR7 co-expression (Klebanoff et al., 2006). As expected, activation induced an expansion of T_CM_ (CD45RA^−^/CCR7^+^) and T_EM_ (CD45RA^−^/CCR7^-^) subsets with a concurrent reduction in T_N_ (CD45RA^+^/CCR7^+^) (Figures 1A and 1B). T_Eff_ (CD45RA^+^/CCR7^-^) were not generated in significant number and were not considered further.

In order to investigate the role of cytokines in determining the fate of recently differentiated memory CD8^+^ T cells, IL-7 was added to the culture medium when the proportion of CD8^+^ T_N_ dropped below 20% of the CD8^+^ population, typically around 1 week after activation (6.7 ± 1.9 days, mean ±1 SD, n = 50). No further activation stimulation was given. In the first 3 days following addition of IL-7 the percentage of CD8^+^ T_N_ continued to diminish, reaching a nadir of 8.4% ± 6.40 (Figure 1B). However, with continuing IL-7 incubation the great majority of CD8^+^ T cells started to re-express CD45RA and reverted back to a phenotype resembling T_N_ and characterized by co-expression of CD45RA, CCR7, CD62L, and CD27 and loss of CD45RO expression (Figures 1A and S1A). We termed these cells that reverted to a naive-like phenotype as “T naive-revertant” (T_Nrev_). This re-acquisition of a naive-like phenotype by CD8^+^ memory T cells reached a plateau by 13–28 days after initial activation (20 ± 4.7 days) and typically represented over 70% of the CD8^+^ T cell population (mean 71% ± 12, range 45%–95%). As such, this value was only slightly below the mean of 87% of CD8^+^ T_N_ at day 0 (±5.8, range 74–98). All samples followed a similar pattern, although there was variation both in the time taken to reach the peak T_Nrev_ level and in the magnitude of the T_Nrev_ population at plateau (Figure 1B). These differences in the percentage of cells with naive phenotype between day 0 and nadir, and between nadir and plateau, were highly significant (p = 1.99 × 10^−50^ and p = 2.69 × 10^−38^, respectively, by paired t test).

To demonstrate that the phenotypic reversion of differentiated T cells was not due to selective death or proliferation of individual T cell subsets, we enumerated the cells within each cell subset and monitored their proliferation. The total number of all cell subsets before and after reversion remained largely unchanged (Table S1), and no cell proliferation was detected after the addition of IL-7 and during the period of phenotypic reversion (Figure 1C). To further confirm that the phenomenon was due to modulation of cellular phenotype, recently differentiated CD8^+^ T_CM_ and T_EM_ were purified, incubated with IL-7, and tracked. Phenotype reversion was again demonstrated for over 80% of the purified T_CM_ and T_EM_ (Figure 1D).

### T_Nrev_ May Undergo Several Rounds of Differentiation and Reversion

We next assessed whether this property was unique to IL-7 or shared by other cytokines. Recently differentiated CBMCs were incubated with single and multiple combinations of the γ-chain cytokines IL-2, IL-7, IL-15, IL-4, and IL-21. IL-6, an inflammatory cytokine, was also incorporated.

Phenotypic reversion was observed with several of these cytokines, but IL-7 was the most potent agent (Figure 1E). Interestingly, CD8^+^ T cells cultured with IL-6 or IL-21 were driven toward a more differentiated phenotype with a substantial increase in T_Eff_ cells. No synergistic effect was observed when IL-7 was administered together with IL-2, IL-4, or IL-15. In addition, the ability of individual cytokines to promote dedifferentiation to T_Nrev_ cells was correlated with their ability to maintain CD8^+^ T cell survival *in vitro* (Figure 1F), whereas IL-6, IL-21, or medium alone led to substantial cell death. This suggests that reversion may be a default physiological program of recently differentiated CD8^+^ T cells when a survival stimulus is provided.

Phenotypic reversion also takes place following differentiation induced by mitogenic stimuli other than soluble anti-CD3 (Figure 1G). The percentage of differentiated CD8^+^ T cells undergoing IL-7-dependent phenotype reversion after activation with phytohemagglutinin (PHA) and staphylococcus enterotoxin B (SEB) was similar to those following activation with anti-CD3. Activation with CD3/CD28 beads led to a smaller proportion of differentiated cells reverting to a naive phenotype, but we were unable to fully remove the beads, some of which were still attached to the cells when IL-7 was added, and it is likely that the resulting continuous antigen stimulation explains the lesser reversion achieved. However, the kinetics of reversion were similar demonstrating that co-stimulation does not prevent phenotypic reversion.

We further assessed whether cells could undergo more than one cycle of phenotypic reversion_._ Since serial rounds of anti-CD3 stimulation led to a high rate of cell death, PHA was used for two further rounds of activation, each followed by IL-7 incubation (Figure 1H). Phenotypic reversion was observed after each cycle of activation and IL-7 treatment, indicating that CD8^+^ T_N_ can undergo repeated cycles of differentiation and reversion.

In order to demonstrate that phenotypic reversion is also possible after activation with cognate antigen, CBMCs were transduced with a gene encoding a T cell receptor (TCR) specific for a peptide from the Epstein-Barr virus (EBV) LMP2 protein (Frumento et al., 2013). Following activation and retroviral transduction, cells acquired a predominantly T_CM_/T_EM_ phenotype but reverted to T_Nrev_ when incubated with IL-7. Cells were then re-challenged with peptide-pulsed autologous dendritic cells (DCs) and underwent differentiation again to CD8^+^ T_EM_ within 5 days (Figure 1I). At this point IL-7 was re-added, and after a further 9 days a second reversion to T_Nrev_ was attained, demonstrating that phenotypic reversion is also possible after stimulation with cognate antigen presented by professional antigen-presenting cells.

### CD8^+^ T_Nrev_ Proliferate and Differentiate Rapidly into Functional Effector Cells following Secondary Stimulation

**Figure 2:**
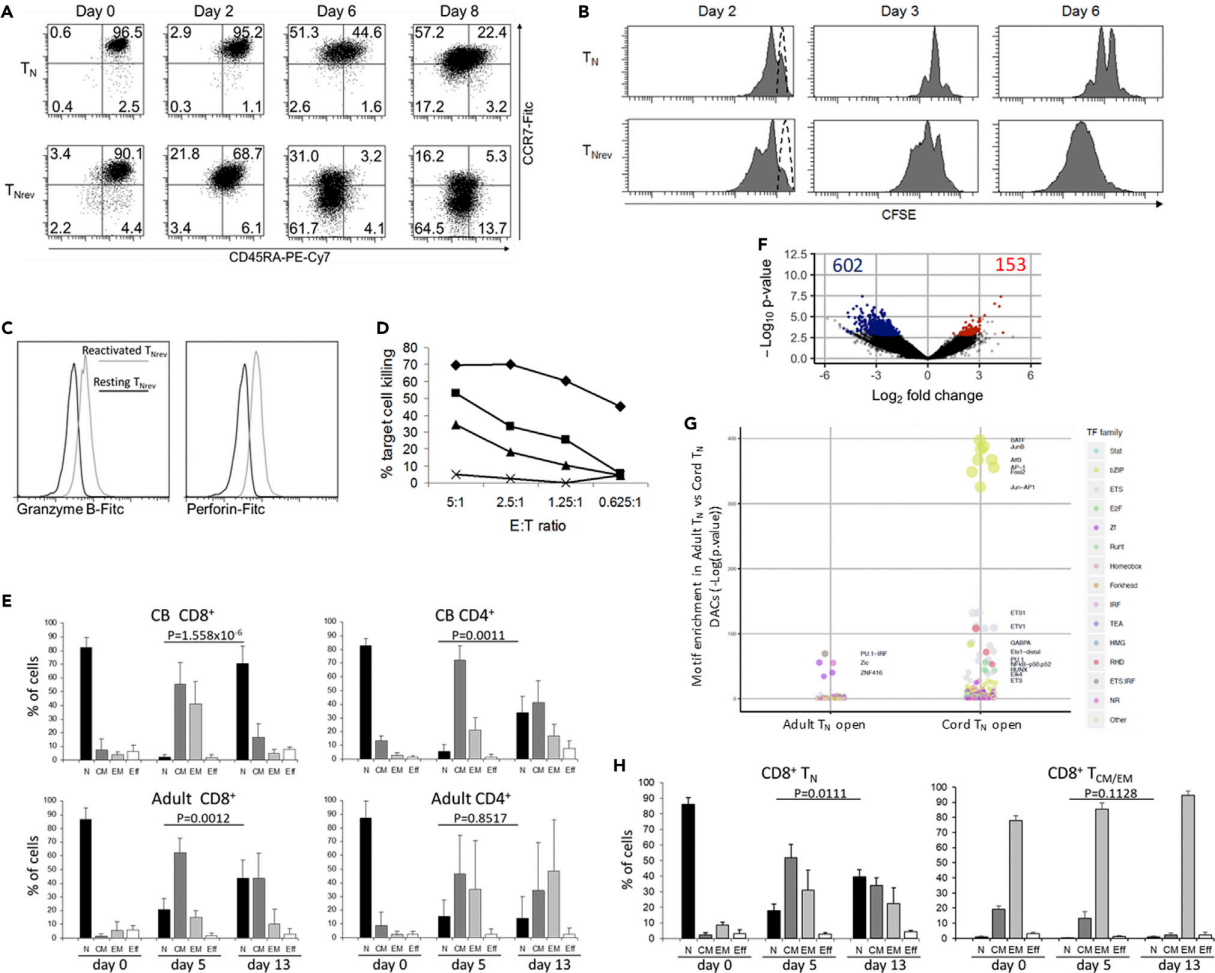
CD8^+^ T_Nrev_ Cells Have Excellent Differentiation and Proliferative Potential and the Amplitude of Reversion Depends on the Cell Source (A) Flow cytometry analysis of phenotype changes in CD8^+^ T_Nrev_ and T_N_ cells from the same CB sample following stimulation with PHA. Single representative experiment out of three. (B) Flow cytometry analysis of the proliferation of CD8^+^ T_Nrev_ and T_N_ cells. Cells from the samples shown in the panel above were stained with CFSE at day 0 and activated with PHA. The CFSE content in the two cell subsets is shown at the indicated time points. Dashed lines represent basal content of CFSE. Single representative experiment out of three. (C) Flow cytometry analysis of perforin and granzyme B expression by the re-stimulated TCR-transduced CD8^+^ T_Nrev_. The intracellular expression of perforin and granzyme B were assessed in T_Nrev_ cells transduced with the SSC-TCR after re-stimulating the cells twice with cognate peptide-pulsed DCs. (D) Cytotoxic assay of re-stimulated, SSC-specific TCR-transduced T_Nrev_. The T cells were incubated in a standard ^51^Cr cytolytic assay with target cells consisting of HLA A∗1101-transduced T2 cells loaded with either 1 μg/mL (diamonds), 10 ng/mL (squares) or 1 ng/mL (triangles) of SSC peptide. The peptide solvent, i.e., DMSO, was used as control (crosses). The percentage of target cell killing at different E:T ratios is indicated. (E) T_N_ cells were isolated from CBMCs and PBMCs, activated with PHA, and then incubated with IL-7. The percentage of cells in the different subsets was measured by flow cytometry at the indicated time points. Data are represented as mean ± 1SD of five CB samples and eight PB samples. Analysis by paired t test. (F) Differential accessibility of peak regions identified in PB T_N_ cells versus CB T_N_ cells. The x axis indicates Log2 fold change, and the y axis indicates –Log10 p value of all peaks. Colored points indicate differentially accessible chromatin sites with inaccessible sites as blue and accessible sites as red. Data are from three CB and three PB samples. (G) TF-binding motif enrichments at DACs more accessible in T_N_ from PB and from CB. Significant (FDR<0.1) pathway enrichments identified within DEG lists from T_Nrev_. (H) CD8^+^ T_N_, and T_CM_ and T_EM,_ were isolated from adult PB, activated with PHA, and incubated with IL-7 from day 5. The percentage of cells in the different subsets was measured by flow cytometry at the indicated time points. Data are represented as mean ± 1SD of three samples. Paired t test.

As T_Nrev_ are antigen-experienced cells that have previously undergone differentiation and expansion we were interested to assess their proliferative potential when compared with primary T_N_. After re-stimulation T_Nrev_ differentiated into memory subsets more rapidly than T_N_ and exhibited a higher proliferation rate (Figures 2A and 2B). T_Nrev_ also rapidly acquired effector function, and when EBV-specific TCR-transduced T_Nrev_ were re-stimulated with peptide-pulsed DCs and driven to a T_EM_ phenotype the cells expressed perforin and granzyme B (Figure 2C) and exerted cytolytic activity against target cells (Figure 2D).

### T Cells from Cord Blood and Adult Donors Differ in Degree of Reversion and Chromatin Accessibility

In order to demonstrate that phenotypic reversion phenomenon is not just restricted to T cells from CB we next enriched CD8^+^ and CD4^+^ T_N_ cells from the blood of adult donors and compared their ability to revert to T_Nrev_ following *in vitro* activation (Figure 2E). Although the vast majority of CB-derived CD8^+^ T cells could be induced to revert to T_Nrev_, this was seen in less than half of naive CD8^+^ T cells from peripheral blood (PB) of adult donors. The reduced degree of reversion in PB cells could not be related to differences in basal phenotype as these were identical in T_N_ cells from CB and PB (Figure S1B). Instead, a remarkable difference was found in the chromatin landscape of T_N_ from the two sources (Figure 2F), which is predicted to influence a range of biological processes (Figure S2A). Compared with T_N_ from PB, T_N_ from CB showed increased accessibility to sites binding transcription factors (TFs), in particular those from the basic region/leucine zipper motif (bZIP) family, of which BATF was the one with the highest number of open binding sites (Figure 2G). Moreover, T_Nrev_ showed more open chromatin regions annotated as regulatory sites for genes involved in TCR signaling (Figure S2B).

Only recently differentiated T_CM_ and T_EM_ were able to undergo reversion and not established T_CM_ (CCR7^+^/CD45RA^−^) and T_EM_ (CCR7^-^/CD45RA^−^) CD8^+^ T cells from adult blood (Figure 2H). This indicates that, despite similar phenotype, the capacity for IL-7-induced dedifferentiation is observed only within recently differentiated T_CM_ and T_EM_ and is relatively less efficient in adult donors.

**Figure 3:**
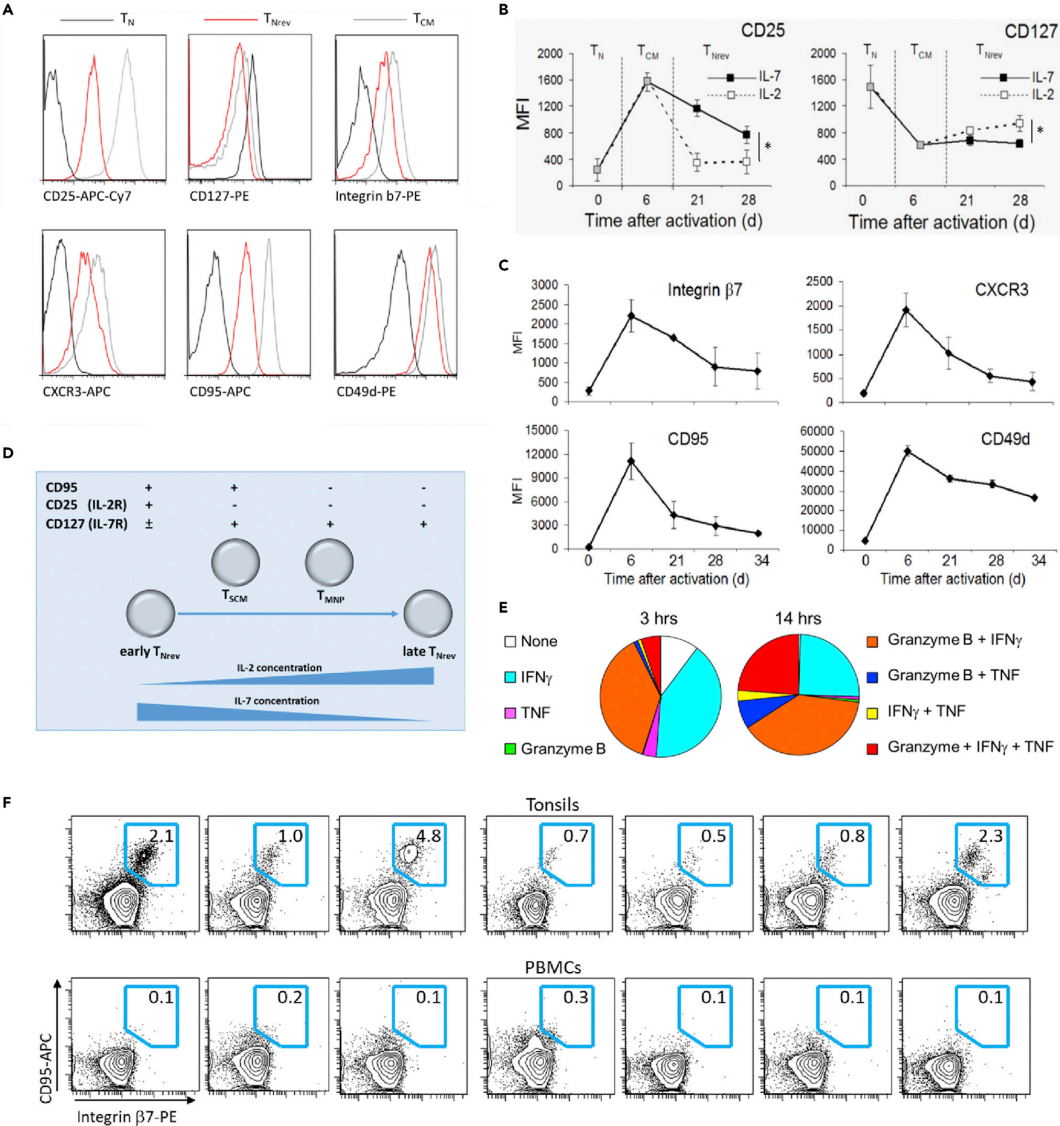
CD8^+^ T_Nrev_ Can Acquire Phenotypic and Functional Characteristics of Other Early Memory T Cell Subsets and Are Present *In Vivo* (A) The expression of the markers that discriminate early T_Nrev_ from T_N_ and recently differentiated T_CM_ was measured by flow cytometry. Single representative experiment out of three. (B) Kinetics of CD25 and CD127 expression by CD8^+^ T_Nrev_ in the presence of either IL-2 or IL-7. After phenotype reversion had occurred, cells were either maintained in 25 ng/mL IL-7 or switched to culture in 30 U/mL IL-2. The absence of one cytokine led to increased expression of its cognate receptor. The mean fluorescence intensity (MFI) is shown. Data are represented as means ± 1SD of three samples. Unpaired t test analysis between the MFI levels at day 28, ∗ = p < 0.05. (C) Kinetics of the expression of discriminatory markers for early T_Nrev_ cells. The MFI was measured at different time points during activation and reversion. Data are represented as means ± 1SD of three samples. (D) Scheme of the differences in phenotype between the memory CD8^+^ T cell subsets showing naive-like phenotype. (E) T_Nrev_ were re-stimulated with PMA plus ionomycin, and the percentages of cells expressing granzyme B, IFNγ, TNF-α, and combinations thereof were measured after 3 and 14 h. Data are represented as mean of three samples. (F) Cells with the T_Nrev_ signature are present among the T_N_ from tonsils, but not from PB. The expression of CD95 and integrin β7 was measured gating CCR7^+^/CD45RA^+^ CD8^+^ T cells from tonsils of seven patients with recurrent acute tonsillitis or from PB of seven unrelated healthy individuals.

### T_Nrev_ Phenotype Overlaps with T_SCM_ and T_MNP_ during *In Vitro* Culture

Using available data (Christensen et al., 2001, Hendriks et al., 2003, Hermiston et al., 2003, Ishida et al., 1992, Kim et al., 2006, O'Shea et al., 1992, Zehnder et al., 1992) we identified 28 membrane-bound proteins that exhibit a differential pattern of expression following T cell activation and differentiation. Antibodies against these proteins were then used to contrast the phenotypic profile of T_N_ and T_Nrev_. The pattern of expression of integrin β7, CD25, CD127, CD95, CXCR3, and CD49d was found to discriminate between these two subsets (Figure 3A). Interestingly, CD95 and CXCR3 are also distinct markers of T_SCM_ (Gattinoni et al., 2011) and CD49d is a marker of T_MNP_ (Pulko et al., 2016). T_Nrev_ and T_CM_ are clearly distinguished by CCR7 and CD45RA expression, but additional differences were also found in the expression of CD25, CD45RO, CD69, CD95, CD120b, CD122, and PTK7 (Figures 3A and S3). These differences demonstrate that the phenotypic correlates of reversion extend substantially beyond differential expression of CCR7 and CD45RA.

We next went on to investigate the relationship between T_Nrev_ cells and T_SCM_ and T_MNP_ subsets. In particular we were interested in the stability of the T_Nrev_ phenotype and how this could be modulated by culture conditions. CD8^+^ T_Nrev_ cells had a very similar phenotype to T_SCM_ and T_MNP_, and all three expressed a common profile of CD45RA^+^/CCR7^+^/CD27^+^/CD62L^+^/CD45RO^−^/CXCR3^+^/CD31^+^/CD122^lo^. Indeed, the only difference between early T_Nrev_ and the other two subsets was that both T_MNP_ and T_SCM_ express the IL-7 receptor (CD127) and T_SCM_ lack expression of the IL-2 receptor (CD25), whereas T_Nrev_ exhibit a CD127^low^CD25^high^ phenotype. However, expression of the receptors for IL-7 and IL-2 is down-regulated on T cells in the presence of their respective cytokines (Minami et al., 1993, Park et al., 2004, Vranjkovic et al., 2007). When T_Nrev_ were deprived of IL-7 and maintained in IL-2 for 2 weeks they adopted a phenotype almost indistinguishable from T_SCM_ and T_MNP_ with rapid decrease in CD25 expression and progressive increase in CD127 expression (Figure 3B). After an additional 2 weeks of culture with IL-2 there was further progressive loss of CD49d, CD95, CXCR3, and integrin β7 (Figure 3C) such that late T_Nrev_ acquire a phenotype approaching that of primary T_N_, although their epigenetic signature clearly identifies them as a different population. We were unable to monitor the cells further due to increased cell death. These results suggest that the cytokine milieu and the time since activation account for the modest phenotypic diversity between T_Nrev_, T_SCM_, and T_MNP_ (Figure 3D). T_Nrev_ also share functional characteristics with T_MNP_ as the latter cells express granzyme B and secrete IFNγ and TNF-α after re-stimulation with phorbol myristate acetate (PMA) plus ionomycin (Pulko et al., 2016). Resting T_Nrev_ also became polyfunctional after the same treatment, r (Figure 3E).

To assess the *in vivo* relevance of phenotypic reversion we also looked for the presence of cells with the T_Nrev_ signature in the blood and secondary lymphoid tissue (tonsils) of adult donors. CD8^+^ T cells with a CCR7^+^CD45RA^+^ naive phenotype and expression of CD95 and integrin β7 were present within tonsil but were not seen in blood (p = 0.017, unpaired t test, Figure 3F). The population of memory T cells within tonsil had a CD95^low^ and integrin β7^−^ phenotype (Figure S4) and was clearly distinguishable from the putative T_Nrev_.

### Substantial Chromatin Reorganization Is Observed during Differentiation from T_N_ to T_EM_ and This Is Partially Retained following Reversion

Epigenetic modifications have a profound regulatory influence on CD8^+^ T cell differentiation and function (Henning et al., 2018, Moskowitz et al., 2017). We next used ATAC-seq to investigate the profile of chromatin landscape remodeling during T cell differentiation and reversion. ATAC-seq analysis was performed on purified T_N_, T_CM_, T_EM_, and T_Nrev_ CD8^+^ populations following *in vitro* culture. ATAC-seq read density profiles at phenotype-defining genes such as *CCR7* and *GZMB* were compatible with lineage-specific expression (Figure S5).

**Figure 4:**
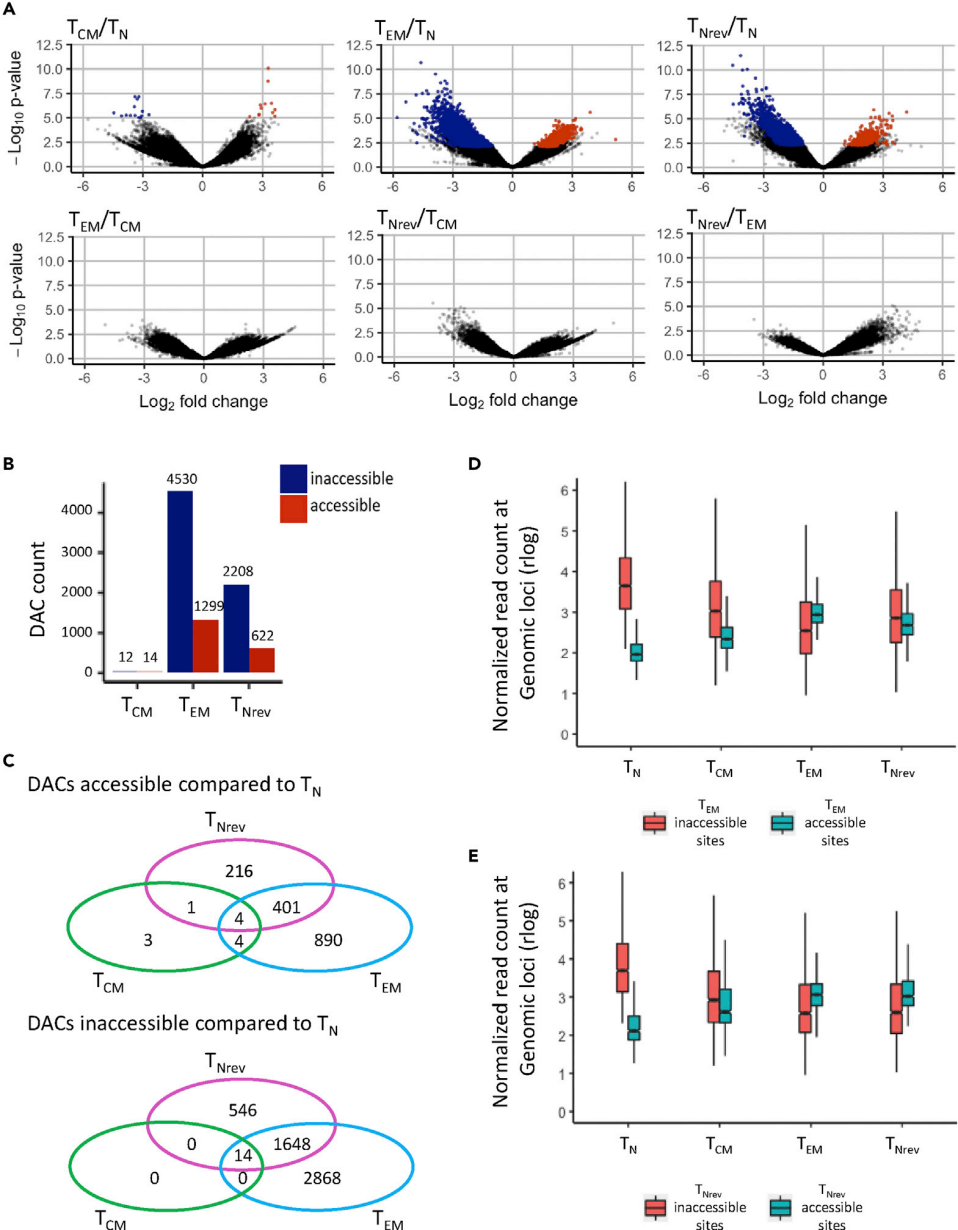
Chromatin Landscape Changes during Differentiation from T_N_ to T_CM_ and T_EM_ and Reversion to T_Nrev_ (A) Pairwise comparisons of chromatin accessibility at peak regions. The x axes indicate Log_2_ fold change, and the y axes indicate unadjusted –Log_10_ p value of all peaks_._ Colored points indicate differentially accessible chromatin sites with inaccessible sites as blue and accessible sites as red. (B) DACs count for accessible and inaccessible regions in comparison with T_N_ are shown for T_CM_, T_EM_, and T_Nrev_. (C) Tracking from T_N_ to T_Nrev_ the accessibility of DACs becoming inaccessible (T_N_ to T_EM_ closing)/accessible (T_N_ to T_EM_ opening) upon differentiation to T_EM_. (D)Tracking from T_N_ to T_Nrev_ the accessibility of DACs becoming inaccessible (T_N_ to T_Nrev_ closing)/accessible (T_N_ to T_Nrev_ opening) upon reversion to T_Nrev._ The y axis is chromatin accessibility in units of rlog normalized mapped reads at the peak site. (E) Overlaps of differentially accessible sites. Overlaps of DACs more accessible in T_CM_/T_EM_/T_Nrev_ compared with T_N_ (left) and DACs less accessible in T_CM_/T_EM_/T_Nrev_ compared with T_N_ (right).

Differentially accessible chromatin sites (DACs) were identified within memory subsets and contrasted with the profile in naive cells (Figures 4A and S6A). The majority of epigenetic modifications were acquired relatively late in differentiation during transition from T_CM_ to T_EM_. In particular, only 26 DACs developed during transition from naive to central memory cells but this increased markedly to 5,829 with further differentiation to T_EM_. Interestingly, 51% of these DACs were lost during reversion to T_Nrev_ but 2,830 DACs still remained within T_Nrev_ cells (Figures 4A and 4B). Read density distributions at T_EM_ and T_Nrev_ DACs further indicate that the chromatin landscape of T_Nrev_ cells retains similarity to T_EM_ cells (Figures 4C and 4D) and is supported by principal component analysis of sample-wise chromatin accessibility, which identified unique groups for the T_N_, T_CM_, and T_EM_ populations and alignment of T_Nrev_ with the memory subtypes (Figure S6B).

We next investigated the balance of open and closed chromatin during differentiation and reversion. A relative increase in the number of inaccessible chromatin regions was observed during differentiation with 4,316 closed and 1,299 open DACs in T_EM_ compared with T_N_ (Figure 4E). This profile was retained within the T_Nrev_ population with values of 2,194 and 617 DACs, respectively. The great majority of DACs within T_Nrev_ were shared with the T_EM_ population, although 216 and 546 regions were uniquely open and closed, respectively, within this subtype.

### Biochemical Pathways Associated with Reversion Can Be Identified by Epigenomic and Transcriptional Analysis of T Cell Subsets

**Figure 5:**
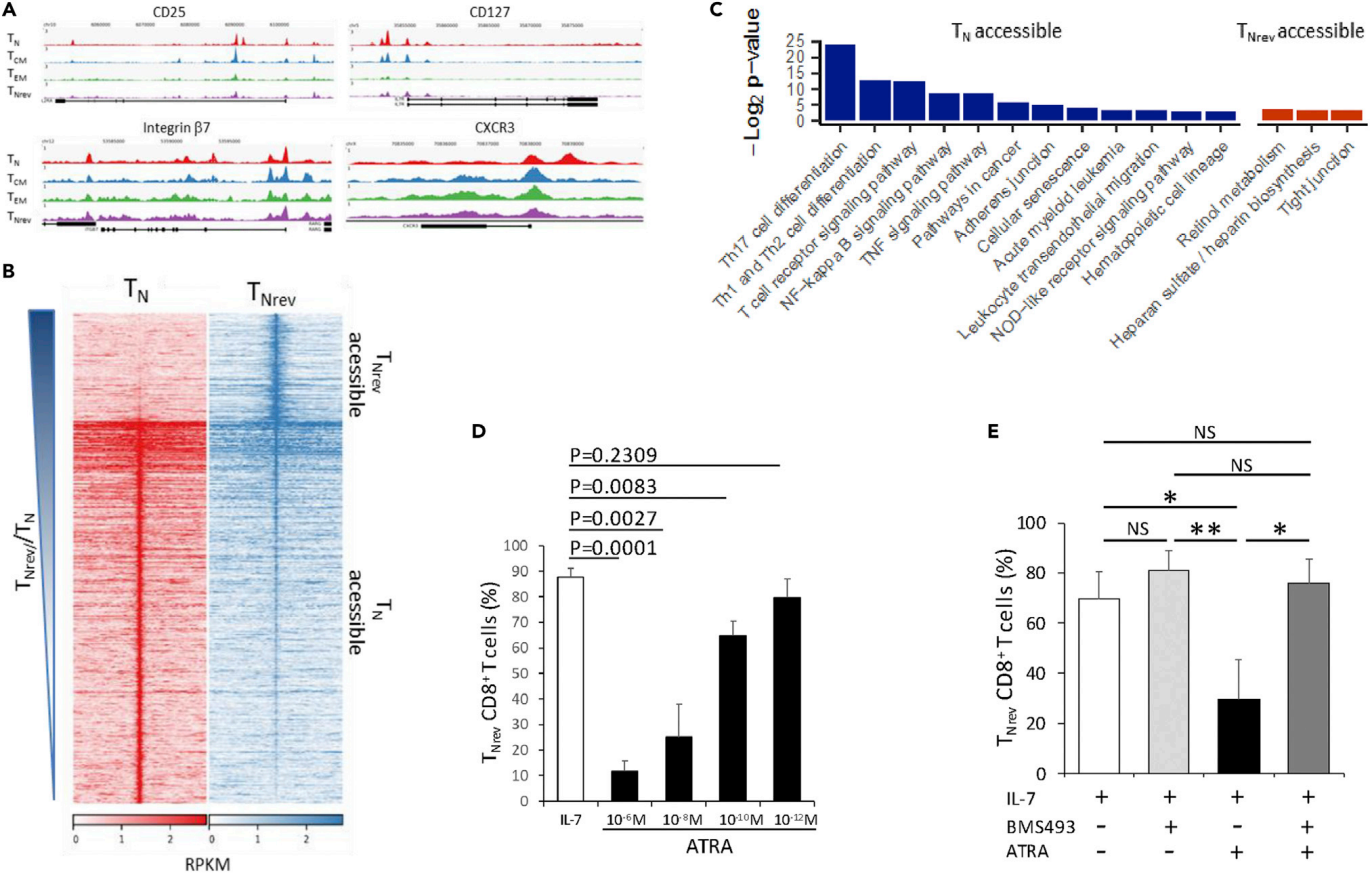
Chromatin Accessibility Identifies Biochemical Pathways Specific for T_N_ and T_Nrev_ (A) ATAC-seq signal tracks at selected markers that discriminate early T_Nrev_ from T_N_ and recently differentiated T_CM_. Gene diagrams (bottom) show alternative transcripts with black boxes indicating exons. Each subset signal is aggregated across the constituent samples, n = 4(T_N_), 3(T_CM_), 3(T_EM_), 4(T_Nrev_). The y axes are in units of reads per million mapped reads. (B) DAC sites in T_Nrev_ versus T_N_ cells. Normalized mapped read density (RPKM) of aggregated T_N_ (n = 4) and T_Nrev_ (n = 4) mapped ATAC-seq reads at differentially accessible chromatin sites (centered on peak summit, extended +/− 5 kbp). (C) Pathways significantly enriched (adjusted p value <0.1) in genes closest to T_N_ accessible or T_Nrev_ accessible DACs. (D) Efficiency of reversion in the presence of ATRA. The percentage of CD8^+^ T_Nrev_ was recorded at day 24 from activation in three CBMC samples. Unpaired t test. (E) After activation CBMCs were incubated with the indicated combination of IL-7 25 (ng/mL), ATRA (10^−8^M), and BMS (493 3 × 10^−6^M). Data are from three CBMC samples. One-way ANOVA.

In order to examine the relationship between chromatin landscape and gene expression, DAC regions were next annotated with the gene whose transcriptional start site was nearest to the peak summit. As anticipated, differential chromatin accessibility was observed at genes encoding phenotypic markers that discriminate T cell subsets including CD25, CD127, integrin β7, and CXCR3 (Figure 5A)_._

Genomic regions enrichment of annotations (GREAT) analysis (Table S2) revealed that the 1,648 DAC sites that became closed in T_EM,_ and were not reopened in T_Nrev_, were enriched for pathways related to TCR signaling in naive T cells and CXCR4-mediated signaling. Sites that were opened during reversion of T_EM_ to T_Nrev_ showed enrichment for IL-7 signal transduction and Lck/Fyn-mediated initiation of TCR activation, whereas the 546 DAC sites uniquely closed in T_Nrev_ were enriched for pathways involved in generation of T cell cytotoxicity.

To further interrogate the differences between true T_N_ and T_Nrev_ cells we combined DAC analysis with transcriptional analysis of the two populations. In total 2,830 DAC regions had been identified between the two subtypes, 622 of which were more accessible in T_Nrev_ and 2,208 less accessible (Figure 5B). These regions were annotated with the gene whose transcriptional start site was nearest to the peak summit. Pathway enrichment analysis with g:Profiler highlighted that genes becoming less accessible in T_Nrev_ included those that regulate the major differentiation pathways for Th1, Th2, and Th17 cells (Figure 5C). Genes associated with retinol metabolism became markedly more accessible in T_Nrev_ and is of note given the pleiotropic effects on retinoic acid on T cell differentiation (Beijer et al., 2013). Indeed, escalating doses of all-trans retinoic acid (ATRA) progressively inhibited reversion of IL-7-treated CD8^+^ T cells (Figure 5D) and this was blocked by a pan-retinoic acid receptor antagonist (Figure 5E).

**Figure 6:**
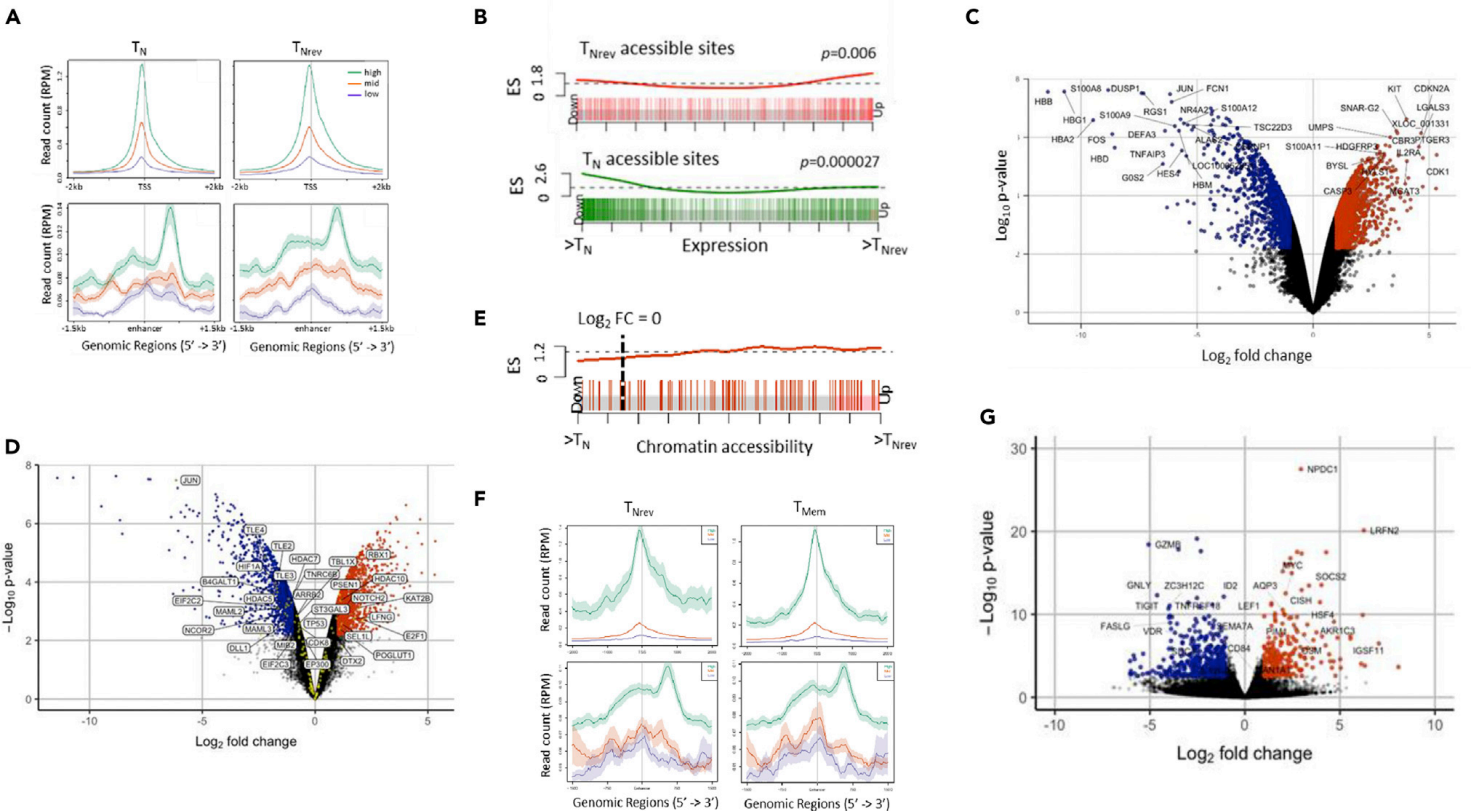
Chromatin Accessibility and Transcriptome Profiles of T_N_ and T_Nrev_ (A) Average mapped read density profiles (centered on TSS (top) and enhancer regions (bottom) generated from aggregated T_N_ (left) and T_Nrev_ (right) ATAC-seq reads. Profiles are for genes identified from transcriptome analysis to be expressed at low, medium, and high levels in T_N_ and T_Nrev_ cells, respectively. (B) Enrichment score (ES) for gene expression changing coordinately with chromatin accessibility. ES of genes closest to T_Nrev_ accessible (red) and T_N_ accessible (green) DACs, vertical bars indicate position of such genes on the axis of fold change in expression (ranking from most down-regulated in T_Nrev_ to most up regulated in T_Nrev_). (C) Microarray analysis was performed on three CD8^+^ T_N_ samples and on the respective CD8^+^ T_Nrev_ cells. Pairwise comparison of gene expression in T_N_ versus T_Nrev_ cells showing –Log_2_ p value versus Log_2_ fold change of all genes_._ Colored points indicate differentially expressed genes that are down-regulated in T_Nrev_ (blue) and up-regulated in T_Nrev_ (orange). (D) The genes in the Notch pathway (yellow dots) that are significantly down-regulated and up-regulated in T_Nrev_ are labeled. (E) Barcode plot from GSEA analysis showing ES of Notch signaling genes within sites ranked by chromatin accessibility. Vertical bars indicate position of such genes on the axis of fold change in chromatin accessibility, ranking from most inaccessible in T_Nrev_ to most up accessible in T_Nrev_. (F) As in (A) but for aggregated T_Nrev_ (left) and T_Mem_ (right) ATAC-seq reads. (G) RNA-seq analysis was performed on four T_Mem_ samples (two T_CM_ and two T_EM_) and on the respective CD8^+^ T_Nrev_ cells. Pairwise comparison of gene expression in T_N_ versus T_Nrev_ cells showing –Log_2_ p value versus Log_2_ fold change of all genes_._ Colored points indicate differentially expressed genes that are down-regulated in T_Nrev_ (blue) and up-regulated in T_Nrev_ (orange).

Transcriptional analysis confirmed that RNA expression levels were strongly associated with ATAC-seq read density at transcription start sites and enhancer regions (Figure 6A), and enrichment analysis of gene sets annotated at T_Nrev_ DACs confirmed strong relationship between chromatin accessibility and expression level (Figure 6B). Transcriptome analysis revealed 2,841 differentially expressed genes including major immune regulators such as *FOS*, *JUN*, *KIT*, and *IL2RA* (Figures 6C and 6F and Table S3). Moreover, 32 of the 104 genes within the Notch signaling pathway were differentially regulated in T_Nrev_ (Figures 6D and 6G). Gene set enrichment analysis (GSEA) of regions closest to Notch signaling genes confirmed an enrichment in accessible chromatin regions in T_Nrev_ cells (Figure 6E) particularly in the *HDAC2* and *HDAC9*-associated regions.

**Figure 7:**
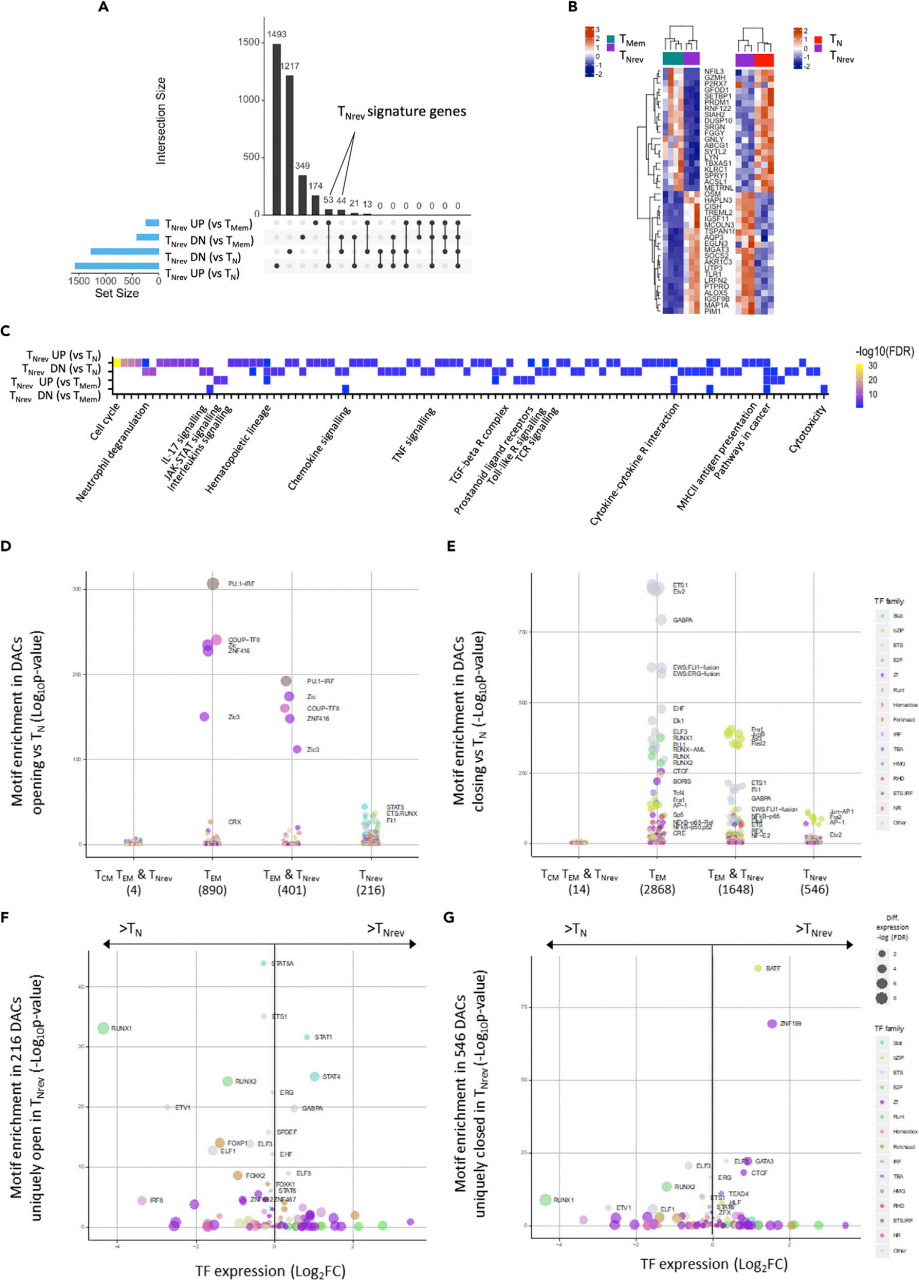
Transcriptional Signatures of T_Nrev_ Cells (A) Intersections of DEGs identified from differential expression analysis of T_Nrev_ versus T_Mem_ and T_Nrev_ versus T_N_. (B) Heatmap of the top 40 T_Nrev_ signature genes. Z-scores were calculated for each gene from RNA-seq (left) and Microarray (right) datasets independently. (C) Significant (FDR<0.1) pathway enrichments identified within DEG lists from T_Nrev_ versus T_Mem_ and T_Nrev_ versus T_N_. (D) TF-binding motif enrichments at DACs more accessible compared with T_N_. Motif enrichments were calculated on the following DACs: 4 shared in T_CM_, T_EM_ and T_Nrev_; 890 in T_EM_; 401 shared in T_EM_ and T_Nrev_; 216 in T_Nrev_. (E) TF-binding motif enrichments at DACs less accessible compared with T_N_. Motif enrichments were calculated on the following DACs: 14 shared in T_CM_, T_EM_, and T_Nrev_; 2,868 in T_EM_; 1,648 shared in T_EM_ and T_Nrev_; 546 in T_Nrev_. (F) Expression of TFs with binding motifs enriched in loci that are uniquely open in T_Nrev_ cells. The x axis indicates fold change in TF expression, and the y axis indicates -log_10_ p value of TF-binding motif enrichment in loci uniquely more accessible in T_Nrev_ cells. Increasing dot size indicates increasingly significant difference in TF expression and color indicates TF family. (G) As in (D) but for TFs with binding motifs enriched in loci uniquely closed after reversion to T_Nrev_.

Comparative transcriptional analysis of T_Nrev_ with memory T cells (T_Mem_) (Figures S7A–S7C) also confirmed RNA expression to be associated with ATAC-seq read density (Figure 6F), and pairwise comparison revealed 447 genes down-regulated in T_Nrev_ including several involved in T cell cytolytic activity such as *GZMB*, *GZMH*, and *GNLY.* In contrast, 267 genes were up-regulated and included proteins that support cell survival and act to block cell differentiation, such as *CISH, NPDC1, HSF4*, and *OSM* (Figure 6G). Interrogation of the transcriptomes of T_Nrev_, T_Mem_, and T_N_ subsets allowed identification of 97 signature genes specific for T_Nrev_ (Figures 7A and 7B) and pathways enriched in genes that were differentially regulated in this subset (Figure 7C). These include a number of intracellular signaling pathways, such as JAK-STAT and the RUNX-dependent regulation of WNT signaling. Comparison of TF transcripts in T_Nrev_ and T_Mem_ (Figure S8) showed differences in the expression of TFs related to stemness and generation of T_SCM_ (Gattinoni et al., 2012, Kondo et al., 2018).

### Reversion Is Associated with Opening of Binding Sites for RUNX and STAT Transcription Factors and Closure of Sites that Bind BATF

TFs can instigate chromatin remodeling and act as important regulators of differentiation. As such, we next assessed the relative enrichment of TF-binding motifs within genomic regions that became differentially open or closed in the different cell subsets. DACs that became more accessible in T_EM_ were enriched in binding motifs for five TF complexes, including PU.1/IRF, COUP-TFII, and zinc finger members, and these open sites were largely retained during reversion to T_Nrev_ cells (Figures 7D and 7E). In contrast, many TF-binding motifs became less accessible upon differentiation to T_EM_ although this profile was not strongly retained in the T_Nrev_ subset.

Finally, in order to gain insight into the potential regulators of reversion we focused on the relative access of TFs for regions of chromatin that were uniquely open or closed in T_Nrev_ cells. Strikingly, chromatin regions containing binding motifs for ET1, RUNX1, and RUNX2, as well as STAT1, STAT4, and STAT5, all became more accessible during reversion, although transcriptional activity from RUNX1 and RUNX2 genes was suppressed in the revertant population (Figure 7F and Table S4). In contrast, there was substantial closure of chromatin regions containing binding motifs for BATF and ZNF189 (Figure 7G and Table S4), which is of particular note given the importance of BATF in driving PD-1-mediated T cell exhaustion (Wherry, 2011).

## Discussion

There is increasing appreciation of the importance of cellular plasticity (Blanpain and Fuch, 2014), and reversion of mature cells into stem cells has been confirmed in some organ systems (Stange et al., 2013, Tata et al., 2013). Here we show that extensive phenotypic reversion of effector CD8^+^ T cells to naive phenotype can occur within the human immune system. Reversion was mediated primarily by IL-7, which is expressed by a range of stromal cells within secondary lymphatic organs and is therefore available to support reversion following local antigen-driven activation (Huang and Luther, 2012). Indeed, cells with a T_Nrev_ phenotype were located within human tonsil. An interesting observation was that the addition of IL-7 markedly decreased apoptosis, suggesting that reversion may be a “default” pathway of recently activated T_N_ cells in the presence of appropriate survival signals (Hong et al., 2012, Mackall et al., 2011, Surh and Sprent, 2008).

The finding sheds light on the potential relationship between the T_SCM_, T_MNP_, and T_Nrev_ populations. These three groups share a common core phenotype exemplified by expression of CD45RA, CCR7, and CXCR3. CD45RA regulates the signaling threshold in T lymphocytes (Hermiston et al., 2003, Irles et al., 2003), and CCR7 permits entry into secondary lymphoid tissue, whereas CXCR3 plays a fundamental role in extravasation of CD8^+^ T cells into inflammatory sites (Cole et al., 1998, Mikucki et al., 2015, Peperzak et al., 2013). The naive-memory phenotype therefore generates an antigen-specific memory pool, which retains the ability to enter lymphoid tissue but has developed heightened responsiveness to re-challenge and may gain rapid access to tissue sites.

Interestingly, the major differences in the phenotype of T_Nrev_, T_SCM_, and T_MNP_ relate to expression of the IL-7 and IL-2 receptors and these were modulated on T_Nrev_ according to the local concentration of IL-2 and IL-7 (Minami et al., 1993, Park et al., 2004, Vranjkovic et al., 2007). T_SCM_ also express CD95^+^, whereas T_MNP_ express CD49d, and we observed that both receptors are down-regulated during prolonged incubation, with CD95 expression falling more rapidly than CD49d expression. As such, we would suggest that the three populations represent a spectrum of naive-memory cells that are generated by reversion and display modest phenotypic diversity that varies according to cytokine concentration and time since activation. Indeed, phenotypic transition of T_EM_ to T_CM_ populations has been observed in murine systems (Wherry et al., 2003), and reversion from T_EM_ to T_CM_ and from these subsets to T_SCM_ was described in patients following hematopoietic stem cell transplant (Cieri et al., 2015).

This work builds on the work of Cieri et al. who previously demonstrated the generation of T_SCM_ from naive cells after T cell engagement in the presence of IL-7. However, in that study the cytokine was included in culture from the start of activation. We certainly do not rule out the possibility that T_MNP_ and T_SCM_ may also be generated by additional mechanisms such as direct differentiation from T_N_ (Cieri et al., 2013, Lugli et al., 2013, Sabatino et al., 2016, Zanon et al., 2017) and CD8^+^ T_SCM_ have also been induced *in vitro* by activation of T cells in the presence of a glycogen synthase-3β inhibitor (Sabatino et al., 2016). Engagement of the Notch pathway in memory T cells also generates T_SCM_-like CD8^+^ and CD4^+^ T cells (Kondo et al., 2017), and we found a number of genes in the Notch pathway to be differently expressed between T_N_ and T_Nrev_ (Figure 6D). Furthermore, IL-7 can further enhance with Notch signaling to induce a T_N_-like phenotype in recently activated CD8^+^ T cells (Kondo et al., 2018). These findings suggest that notch engagement and IL-7 may represent two alternative mechanisms to generate T_SCM_-like cells.

Importantly, we observed that T_Nrev_ cells were able to undergo several rounds of reversion *in vitro*, and as such this process may be important in protection from both acute and chronic infectious agents. Indeed, it is now clear that a substantial proportion of antigen-experienced T cells is contained within the apparent “naive” CD8^+^ T cell pool (Ellefsen et al., 2002, Remmerswaal et al., 2012). Longer-term culture of T_Nrev_ led to progressive down-regulation of integrin β7 and CXCR3, but it is uncertain if they can ultimately acquire a phenotype indistinguishable from that of primary T_N_.

Most of our work was performed on T cells from CB, which contains very few memory or effector cells. We did observe phenotypic reversion using T_N_ cells from adult donors, although the relative proportion of cells that underwent reversion was sharply reduced and may reflect the decreased number of open chromatin sites and binding sites for TF in adult PB. Interestingly, the epigenetic landscape of CD8^+^ T cells in older people is globally biased toward a differentiated phenotype (Moskowitz et al., 2017) with a reduction in chromatin accessibility that is most apparent at the IL-7R locus (Ucar et al., 2017).

Phenotypic reversion may help to explain the paradox of preservation of the human T_N_ pool during aging despite *in vivo* analyses and mathematical modeling suggesting that thymic output is insufficient for its maintenance (den Braber et al., 2012, Hakim et al., 2005, Murray et al., 2003). Interestingly, it was found that the T_SCM_/T_N_ ratio in PB increases with age (Li et al., 2019), suggesting that during aging naive-memory cells replace T_N_ and sustain immunological memory.

Epigenetic regulation maintains the equilibrium between self-renewal and differentiation of stem cells and regulates tissue homeostasis throughout life. As such we were keen to understand how the chromatin landscape was modified following T cell activation and if these changes were reversible during phenotypic reversion to T_Nrev_. Interestingly, this was largely not the case and T_Nrev_ cells retained an epigenetic profile that was similar to effector cells. As such, T_Nrev_ become “epigenetically primed” for secondary activation at the same time as they undergo phenotypic reversion to a naive-memory phenotype. The chromatin modifications within T_Nrev_ are likely to explain their ability to rapidly differentiate and acquire effector function in response to secondary stimulation and reveal a discrepancy between the degree of phenotypic and epigenetic reversion. This may partly explain the decrease in chromatin accessibility in phenotypically naive CD8^+^ T cells in older people (Ucar et al., 2017). Furthermore, the observation that the chromatin landscape of T_Nrev_ is much more extensively modified than that of T_CM_ provides further confirmation that they have undergone sequential differentiation and reversion rather than minimal differentiation from T_N_.

Analysis of the distribution of TF-binding sites within differentially accessible chromatin regions can help to identify potential transcriptional regulators of differentiation. A striking observation was that many chromatin regions containing binding sites for BATF became closed during reversion. BATF is an essential regulator of CD8^+^ differentiation (Kurachi et al., 2014), and PD-1 engagement on T cells can drive BATF-dependent terminal differentiation (Quigley et al., 2010).

Reversion may play a potential role in limiting T cell exhaustion in both physiological and pathological settings, and it is noteworthy that the chromatin region containing the *IL-7R* gene becomes poorly accessible in exhausted CD8^+^ cells (Scott-Browne et al., 2016). In contrast, chromatin regions enriched for binding sites of several TFs, including RUNX, STAT, and ETS family members, became more accessible within revertant subsets. Stat1 and Stat4 signaling regulate T cell responses to interferon and cytokine signaling (Gil et al., 2006, Nguyen et al., 2002, Thierfelder et al., 1996), STAT5 is critical in maintaining effector CD8 T cell responses (Tripathi et al., 2010), and over-representation of ETS motifs in chromatin accessible regions has been observed previously in naive T cells (Moskowitz et al., 2017). As such the “epigenetic priming” of naive-memory subsets appears to reflect an increased sensitivity to interferon and cytokines within the local microenvironment but protection from terminal differentiation.

Cytokine-driven reversion of recently activated CD8^+^ T cells thus uncovers a novel pathway for T cell differentiation and provides a unifying hypothesis for the existence of a naive-memory pool that contains T_SCM_, T_MNP_, and T_Nrev_ populations. We also show that the chromatin structure of naive-revertant cells is substantially reorganized in comparison with the naive pool and as such they are epigenetically “primed for secondary activation. These observations will help to guide studies of fundamental mechanisms that regulate T cell differentiation and should also be of considerable value for optimal generation of naive-memory cells for adoptive T cell immunotherapy as less differentiated cells have been associated with superior engraftment, persistence, and antitumor activity (Hinrichs et al., 2011, Klebanoff et al., 2011).

### Limitations of the Study

A few limitations should be considered when interpreting our data. Although we identified cells with the phenotype of T_Nrev_ cells in human tonsil, it will be of interest to assess further if these are generated directly *in vivo*. In addition, it will be important to pursue parallel studies within animal models to interrogate potential mechanisms of phenotypic reversion.

## Methods

All methods can be found in the accompanying Transparent Methods supplemental file.

## Data and Code Availability

All relevant data are available from the authors upon request. RNA sequencing data have been deposited in Gene Expression Omnibus, accession number GSE114812.

## Supplemental Information

Document S1. Transparent Methods, Figures S1–S8, Tables S1, S2, and S5

Table S3. Genes Showing Statistically Significant Difference in the Levels of Expression between T_N_ and T_Nrev_, Related to Figure 6

Table S4. Transcription Factor Binding Motifs Enriched in Differentially Accessible Genomic Regions, Related to Figure 7
