# Supplementary material for: Homeostatic Cytokines Drive Epigenetic Reprogramming of Activated T Cells into a “Naive-Memory” Phenotype
Source: iScience. 2020 Mar 19;23(4):100989. doi: 10.1016/j.isci.2020.100989 (PMC7115140; doi:10.1016/j.isci.2020.100989)
Supplement: Document S1. Transparent Methods, Figures S1–S8, Tables S1, S2, and S5 [file mmc1.pdf]

iScience, Volume *23*

## Supplemental Information

# Homeostatic Cytokines Drive Epigenetic Reprogramming of Activated T Cells into a “Naive-Memory” Phenotype

**Guido Frumento, Kriti Verma, Wayne Croft, Andrea White, Jianmin Zuo, Zsuzsanna Nagy, Stephen Kissane, Graham Anderson, Paul Moss, and Frederick E. Chen**

SUPPLEMENTAL INFORMATION

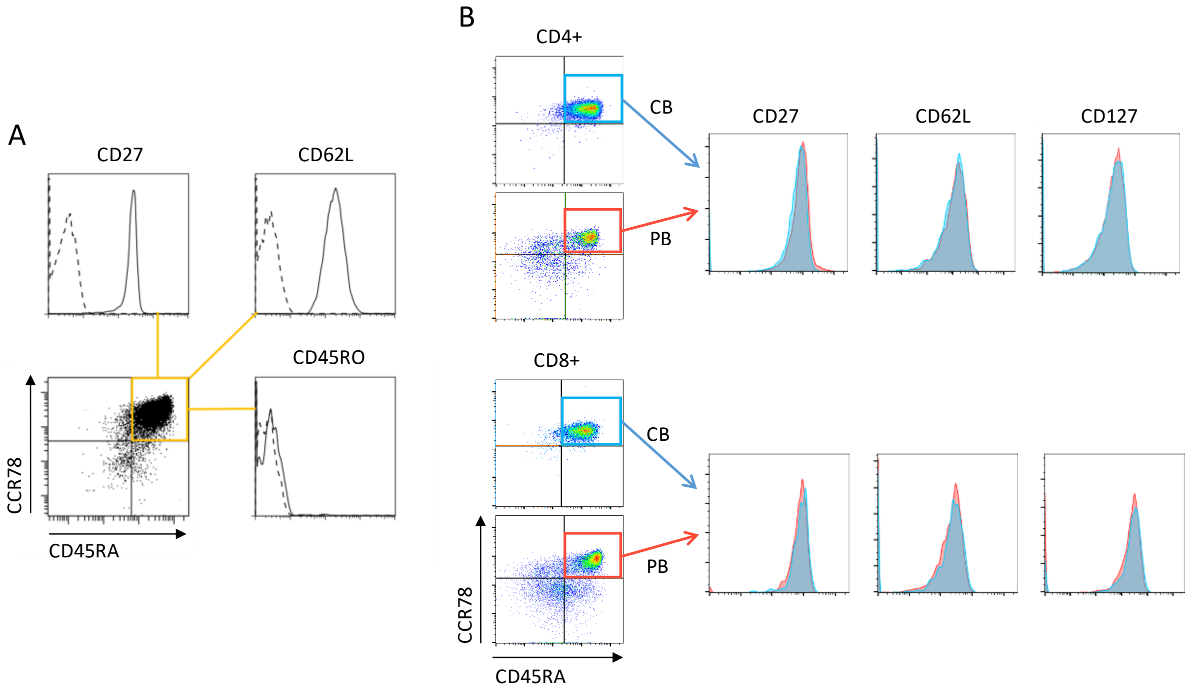


**Figure S1. The basal phenotype of $T_N$ and $T_{Nrev}$ from CB and PB, Related to Figures 1 and 2.** (A) The expression of CD27, CD45RO and CD62L in $T_{Nrev}$. The expression is shown for the cells having undergone phenotype reversion in Figure 1A, right panel. Dashed lines show the profiles of isotype controls. (B) The basal phenotype of $T_N$ from CB and PB is shown.

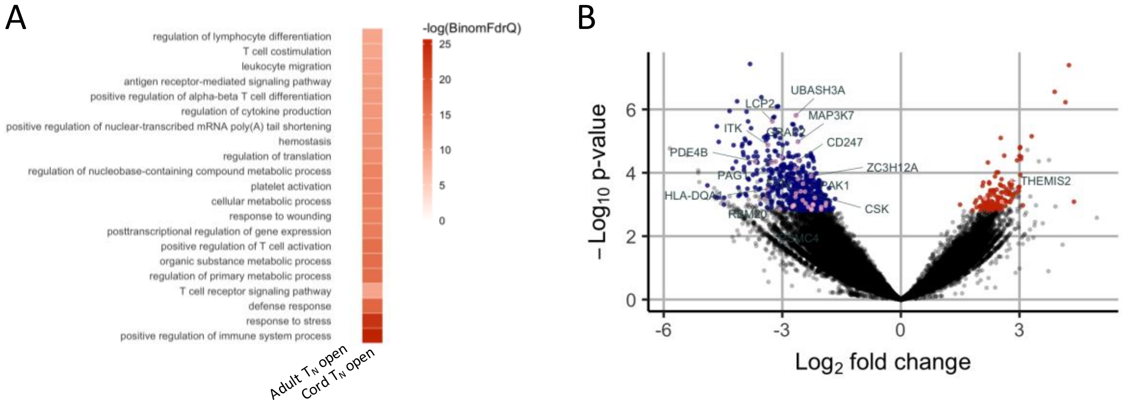


**Figure S2. Pathways significantly enriched in chromatin sites preferentially open in $T_N$ from PB or CB, Related to Figure 2.** (A) Selected significant (FDR<0.1) enrichments of GO terms (Biological Process) from DAC-associated gene annotations for DAC sites open in Cord TN cells. No significant functional enrichments could be found at the sites preferentially open in samples from adults. Results are from 3 CB and 3 PB samples. (B) . Differential accessibility of peak regions identified in PB $T_N$ cells vs CB $T_N$ cells. The x-axes indicates Log2 fold change and the y-axes indicates –Log10 p-value of all peaks. Colored points indicate differentially accessible chromatin sites with inaccessible sites as blue and accessible sites as red. Points in pink are differentially accessible regions annotated as regulatory sites for genes in the biological process GO term T cell receptor signalling.

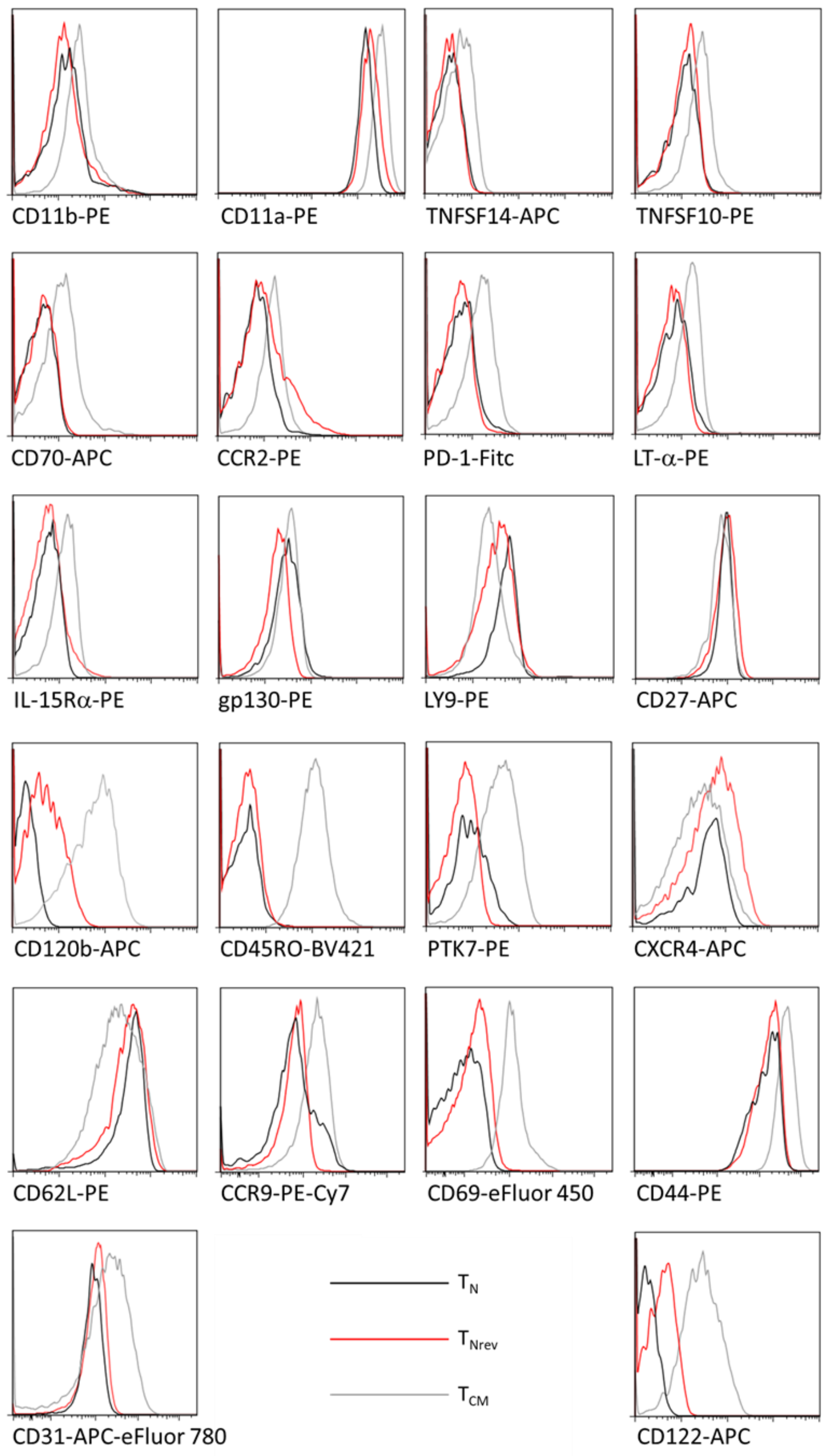


**Figure S3. Extended screening for putative markers of $T_{Nrev}$, related to Figure 3.** The membrane expression of non-discriminatory markers in $T_N$, recently reverted $T_{Nrev}$ and recently differentiated $T_{CM}$ is shown. Single representative experiment out of three.

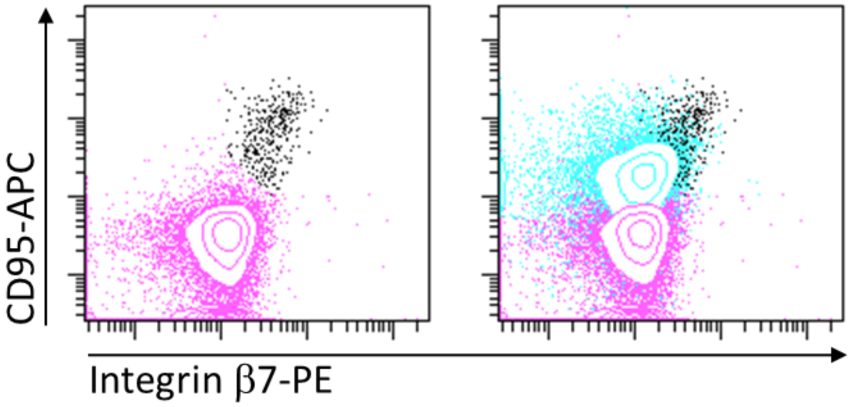


**Figure S4. In tonsils, putative $T_{Nrev}$ and memory $CD8^+$ T cells differ in the expression of CD95 and integrin β7, Related to Figure 3.** In the left panel the expression of the two markers is shown for $CCR7^+$/$CD45RA^+$ $CD8^+$ T cells; in the right panel the expression of CD95 and integrin β7 in memory $CD8^+$ T cells, either $CCR7^+$/$CD45RA^-$, $CCR7^-$/$CD45RA^-$ or $CCR7^-$/$CD45RA^+$, is superimposed. In purple the classical $T_N$, in black the putative $T_{Nrev}$, and in pale blue the memory cells. Single representative experiment out of seven.

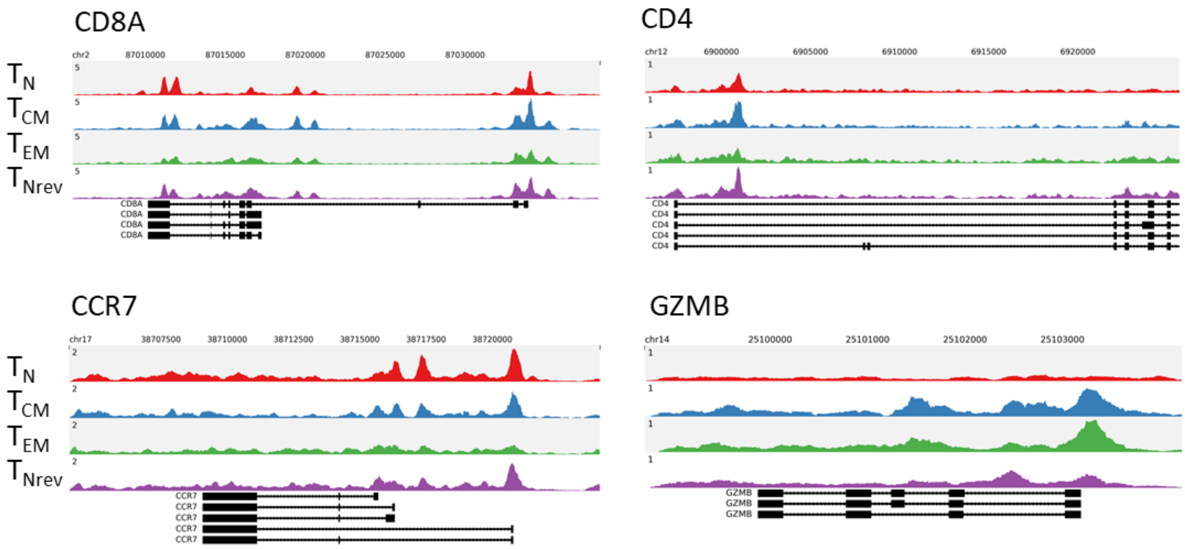


**Figure S5. ATAC-seq signal tracks at selected genes confirming the purity of the samples, related to Figure 4.** Gene diagrams (bottom) show alternative transcripts with black boxes indicating exons. Each subset signal is aggregated across the constituent samples, n = 4($T_N$), 3($T_{CM}$), 3($T_{EM}$), 4($T_{Nrev}$). The y-axes are in units of reads per million mapped reads.

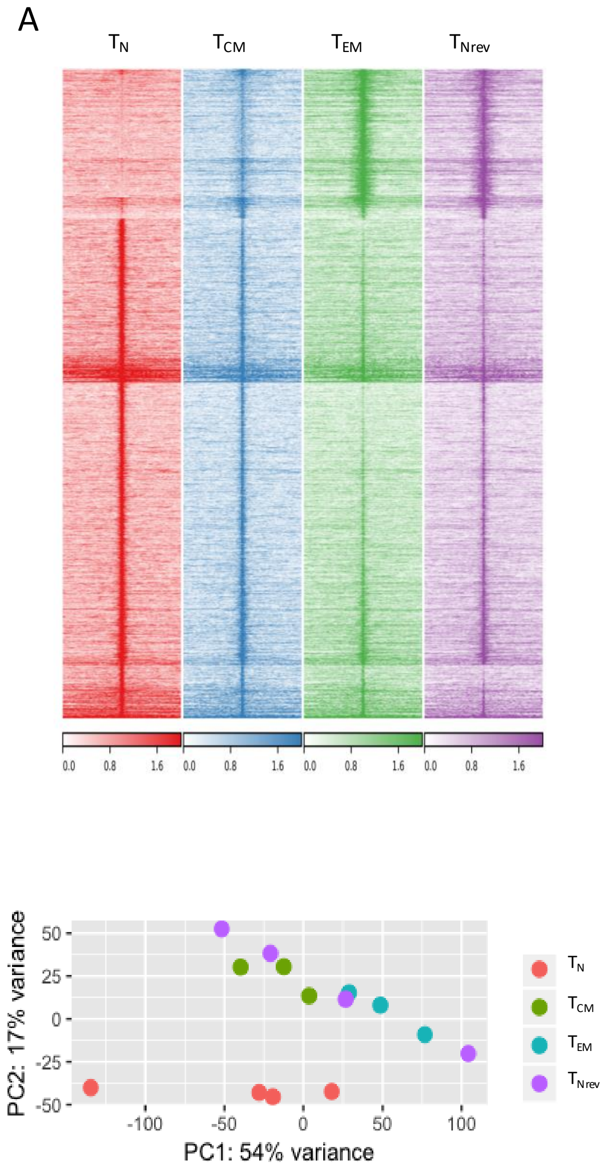


**Figure S6. Comparison of chromatin landscape in $T_N$, $T_{CM}$, $T_{EM}$ and $T_{Nrev}$, Related to Figure 4**. (A) Peak sites that were differentially accessible in at least one pairwise comparison. Shown are normalized mapped read density (RPKM) of aggregated $T_N$ (n=4), $T_{CM}$ (n=3) , $T_{EM}$ (n=3) and $T_{Nrev}$ (n=4) mapped ATAC-seq reads at differentially accessible chromatin sites (centred on peak summit, extended +/- 5kbp). (B) DACs counts for accessible and inaccessible sites are shown for $T_{CM}$, $T_{EM}$ and $T_{Nrev}$ in comparison to $T_N$.

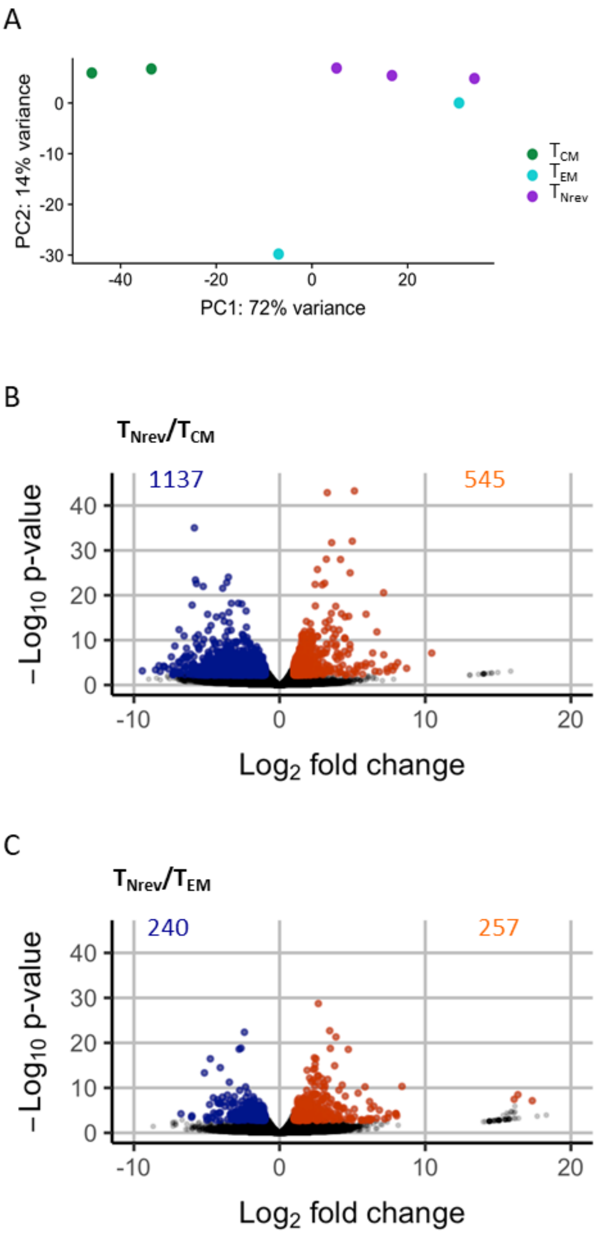


**Figure S7. Transcriptomes of $T_{Nrev}$ compared $T_{Mem}$ cells, Related to Figure 6.** Gene expression analysis was performed by RNAseq on 3 CD8$^+$ $T_{Nrev}$ samples, and 4 CD8$^+$ $T_{Mem}$ (2 $T_{CM}$ and 2 $T_{EM}$) samples. (A) PCA plot of all samples. Pairwise comparisons of gene expression in $T_{Nrev}$ vs $T_{CM}$ cells (B) and $T_{Nrev}$ vs $T_{EM}$ (C) show –Log10 p-value vs Log2 fold change of all genes. Colored points indicate differentially expressed genes that are down-regulated (blue) and up-regulated (orange) in $T_{Nrev}$.

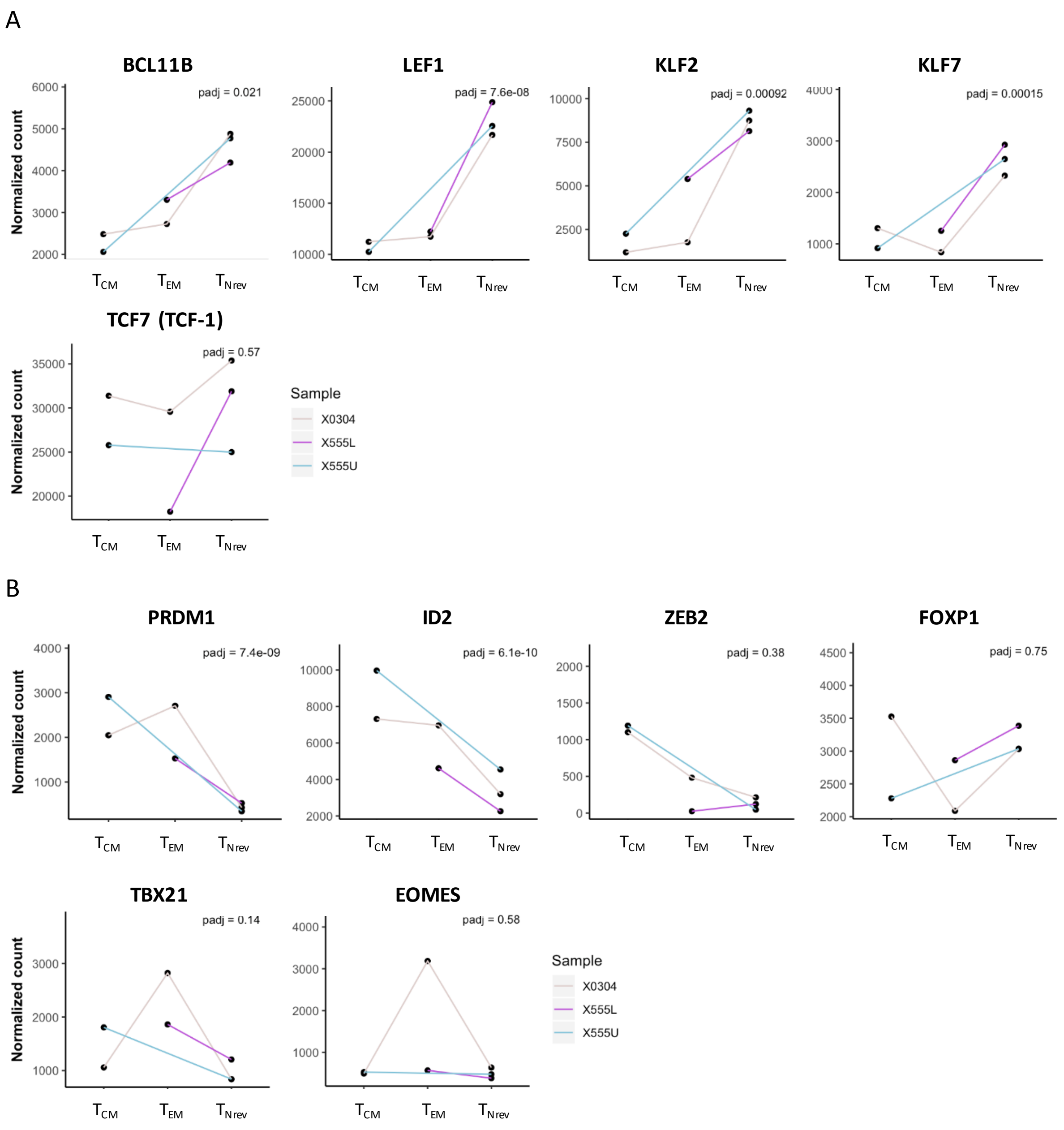


**Figure S8. Stemness transcription factors of interest, Related to Figure 7**. Normalized expression from RNA-seq of $T_{CM}$, $T_{EM}$ and $T_{Nrev}$ cells. Padj = p value adjusted for multiple testing from differential expression analysis of $T_{Nrev}$ vs $T_{CM}$ and $T_{EM}$ cells. Key Transcription factors with known increased (A) and decreased (B) expression.

Table S1. Cell numbers are not altered by phenotypic reversion, Related to Figure 1.

| | CB 1 | | CB 2 | | CB 3 | |
|---|---|---|---|---|---|---|
| | **Day6** | **Day 16** | **Day8** | **Day 18** | **Day6** | **Day 20** |
| $CCR7^+/CD45RA^+$ | 32,1[A] | 872,1 | 9 | 345,5 | 21,3 | 1769,3 |
| $CCR7^+/CD45RA^-$ | 982,2 | 256,6 | 782,2 | 303,2 | 1560,9 | 487,9 |
| $CCR7^-/CD45RA^-$ | 308 | 79,2 | 183,7 | 101,4 | 213,2 | 33,1 |
| $CCR7-/CD45RA^+$ | 5,4 | 72 | 1,4 | 32 | 2,9 | 43 |
| Total | 1327,7 | 1279,9 | 976,3 | 782,1 | 1798,3 | 2333,3 |

[A] Absolute number of CD8+ T cells within different subsets at $T_N$ nadir (days 6 and 8) and $T_{Nrev}$ plateau (days 16, 18 and 20). Cell counts ($x10^3$) from 3 different CB samples, enumerated using Trucount beads.

Table S2. GREAT analysis, Related to Figure 5.

| | | | |
|---|---|---|---|
| **216 TNrev accessible sites** | | | |
| | NO ENRICHMENT | | |
| **401 TNrev and TEM accessible sites** | | | |
| | NO ENRICHMENT | | |
| **890 TEM accessible sites** | | | |
| | NO ENRICHMENT | | |
| **546 TNrev inaccessible sites** | | | |
| | **Binom Rank** | **Binom Raw P-Value** | **Binom FDR Q-Val** |
| Signaling events mediated by focal adhesion kinase | 2 | 2.16441E-06 | 0.001428513 |
| Signaling events mediated by Hepatocyte GF Receptor (c-Met) | 3 | 2.50412E-06 | 0.001101811 |
| T Cytotoxic Cell Surface Molecules | 5 | 1.64991E-05 | 0.004355768 |
| Keratinocyte Differentiation | 7 | 0.000126032 | 0.023766053 |
| PDGFR-beta signaling pathway | 10 | 0.000228715 | 0.03019034 |
| HIF-1-alpha transcription factor network | 15 | 0.000471389 | 0.041482267 |
| **1648 TNrev and TEM inaccessible sites** | | | |
| | **Binom Rank** | **Binom Raw P-Value** | **Binom FDR Q-Val** |
| TCR signaling in naive CD4+ T cells | 1 | 2.46E-27 | 3.24E-24 |
| Genes involved in Immune System | 2 | 3.81E-26 | 2.51E-23 |
| TCR signaling in naive CD8+ T cells | 3 | 1.18E-22 | 5.18E-20 |
| T cell receptor signaling pathway | 4 | 4.57E-21 | 1.51E-18 |
| Thromboxane A2 receptor signaling | 5 | 2.33E-18 | 6.14E-16 |
| Genes involved in Generation of second messenger molecules | 6 | 3.36E-17 | 7.40E-15 |
| Keratinocyte Differentiation | 7 | 5.08E-17 | 9.58E-15 |
| CXCR4-mediated signaling events | 8 | 7.31E-17 | 1.21E-14 |
| Genes involved in TCR signaling | 9 | 2.35E-15 | 3.44E-13 |
| Genes involved in Adaptive Immune System | 10 | 2.23E-14 | 2.94E-12 |
| T Cell Signal Transduction | 11 | 7.59E-14 | 9.10E-12 |
| Genes involved in Platelet activation, signaling and aggregation | 12 | 5.48E-13 | 6.03E-11 |
| HIF-1-alpha transcription factor network | 13 | 2.49E-11 | 2.53E-09 |
| Signaling events mediated by VEGFR1 and VEGFR2 | 14 | 5.20E-11 | 4.90E-09 |
| Genes involved in Cytokine Signaling in Immune system | 15 | 1.64E-10 | 1.44E-08 |
| Signaling events mediated by focal adhesion kinase | 16 | 2.42E-10 | 2.00E-08 |
| Genes involved in Lipoprotein metabolism | 17 | 3.53E-10 | 2.74E-08 |
| Role of Calcineurin-dependent NFAT signaling in lymphocytes | 19 | 7.14E-10 | 4.96E-08 |
| Genes involved in Lipid digestion, mobilization, and transport | 21 | 7.27E-10 | 4.57E-08 |
| Genes involved in Signaling by NOTCH | 22 | 9.66E-10 | 5.80E-08 |
| **2868 TEM inaccessible sites** | | | |
| | **Binom Rank** | **Binom Raw P-Value** | **Binom FDR Q-Val** |
| Genes involved in Adaptive Immune System | 2 | 1.11E-23 | 7.36E-21 |
| TCR signaling in naive CD4+ T cells | 3 | 1.88E-23 | 8.28E-21 |
| TCR signaling in naive CD8+ T cells | 4 | 1.89E-21 | 6.24E-19 |
| T cell receptor signaling pathway | 5 | 9.77E-20 | 2.58E-17 |
| CXCR4-mediated signaling events | 6 | 8.47E-19 | 1.86E-16 |
| Class I PI3K signaling events | 7 | 1.36E-17 | 2.57E-15 |
| Genes involved in TCR signaling | 8 | 8.07E-16 | 1.33E-13 |
| Natural killer cell mediated cytotoxicity | 9 | 4.53E-15 | 6.64E-13 |
| Genes involved in Cytokine Signaling in Immune system | 10 | 8.55E-15 | 1.13E-12 |
| T Cell Receptor Signaling Pathway | 11 | 7.90E-14 | 9.48E-12 |
| Genes involved in Signaling by the B Cell Receptor (BCR) | 12 | 8.12E-14 | 8.94E-12 |
| Genes involved in Phosphorylation of CD3 and TCR zeta chains | 13 | 6.23E-13 | 6.33E-11 |
| IL2-mediated signaling events | 15 | 2.12E-12 | 1.86E-10 |
| Signaling events mediated by focal adhesion kinase | 16 | 3.08E-12 | 2.54E-10 |
| Lck and Fyn tyrosine kinases in initiation of TCR Activation | 17 | 6.43E-12 | 4.99E-10 |
| PDGFR-beta signaling pathway | 18 | 6.77E-12 | 4.97E-10 |
| IL-7 Signal Transduction | 19 | 3.86E-11 | 2.68E-09 |
| Downstream signaling in naive CD8+ T cells | 20 | 4.83E-11 | 3.19E-09 |
| Thromboxane A2 receptor signaling | 21 | 9.06E-11 | 5.69E-09 |
| Fc-epsilon receptor I signaling in mast cells | 22 | 1.15E-10 | 6.87E-09 |

Table S5. List of the antibodies used for flow cytometry, Related to Figure 1, Figure 2, Figure 3, Figure 5.

| Antigen/ fluorochrome | Clone | Purchased from | Antigen/ fluorochrome | Clone | Purchased from |
|---|---|---|---|---|---|
| CCR2/PE | K036C2 | BioLegend | CD45RA/Fitc | HI100 | BD |
| CCR7/Fitc | G043H7 | BD | CD45RA/PE-Cy7 | HI100 | BD |
| CCR7/PE | G043H7 | R&D | CD45RO/BV421 | UCHL1 | BioLegend |
| CCR7/BV605 | G043H7 | BioLegend | CD49d/PE | 9F10 | eBioscience |
| CCR9/PE-Cy7 | L053E8 | BioLegend | CD62L/PE | DREG-56 | BD |
| CD11a/PE | HI111 | BD | CD69/eFluor 450 | FN50 | eBioscience |
| CD11b/PE | ICRF44 | BD | CD70/APC | 113-16 | BioLegend |
| CD120b/APC | 22235 | R&D | CD8/Fitc | SK1 | BD |
| CD122/APC | TU27 | BioLegend | CD8/Pacific Blue | RPA-T8 | BD |
| CD127/PE | A019D5 | BioLegend | CD95/APC | DX2 | BD |
| CD14/Pacific Blue | TuK4 | Invitrogen | CXCR3/APC | 1C6/CXCR3 | BD |
| CD14/PerCP | 134620 | R&D | CXCR4/APC | 12G5 | eBioscience |
| CD16/Pacific Blue | 3G8 | Invitrogen | gp130/PE | 28126 | R&D |
| CD16/PerCP | 245536 | R&D | granzyme B/Fitc | GB11 | BD |
| CD19/Pacific Blue | SJ25-C1 | Invitrogen | IL-15Ra/PE | JM7A4 | BioLegend |
| CD19/PerCP | 4G7-2E3 | R&D | Integrin $\beta$7/PE | 473207 | R&D |
| CD25/APC-Cy7 | M-A251 | BD | LT-$\alpha$/PE | 359-81-11 | BioLegend |
| CD27/APC | 57703 | R&D | LY-9/PE | hlY-9.1.25 | BioLegend |
| CD3/PE | OKT3 | BioLegend | PD-1/Fitc | MIH4 | BD |
| CD3/V500 | UCHT1 | BD | Perforin/Fitc | Dg9 | eBioscience |
| CD31/APC-eFluor 780 | WM-59 | eBioscience | PTK7/PE | 188B | Miltenyi |
| CD4/Fitc | SK3 | eBioscience | TNFSF10/PE | RIK-2 | BioLegend |
| CD44/PE | BJ18 | BioLegend | TNFSF14/APC | 115520 | R&D |

## TRANSPARENT METHODS

### Cell separation and culture.

The study was approved by the National Research Ethics Committee, UK REC no. 11/WM/0315, and by the Non-Clinical Issue committee of the NHS Blood and Transplant. Human CB from anonymized collections unsuitable for banking, was provided by the NHS Cord Blood Bank, UK, as Non-Clinical Issue. Peripheral blood (PB) was collected from consenting adult healthy blood donors from the NHS Blood and Transplant Donor Centre, Birmingham, UK.

PB mononuclear cells (PBMCs) and CBMCs were obtained by Ficoll separation. $CD8^+$ $T_N$ and $T_{Nrev}$ were enriched using the Naïve $CD8^+$ T cell Isolation Kit (Miltenyi Biotech, Bergisch Gladbach, Germany). $CD8^+$ $T_{EM}$ cells were negatively isolated after activation of enriched $CD8^+$ $T_N$ by removal of $CCR7^+$ and $CD45RA^+$ cells with anti-CCR7-APC, anti-CD45RA-APC and anti-APC MicroBeads (Miltenyi). $CD8^+$ $T_{CM}$ cells were isolated from less differentiated samples by depletion of $CD45RA^+$ cells with anti CD45RA-APC and anti-APC MicroBeads. Cells were cultured in RPMI 1640 plus 10% FCS. $CD4^+$ $T_N$ cells were isolated with the Naïve $CD4^+$ T cell Isolation Cell Isolation Kit II (Miltenyi).

$CD8^+$ $T_{CM/EM}$ cells were negatively isolated from $CD8^+$ $T_N$ cells isolated as indicated above by removal of $CD45RA^+$ cells with anti-CD45RA-APC and anti-APC MicroBeads (Miltenyi).

Dendritic cells (DCs) were generated from adherent mononuclear cells after incubating in plates for 2 hours. Adherent cells were cultured in the medium supplemented on days 0, 3 and 6 with 50 ng/ml GMCSF and 500 U/ml IL-4. On day 6, DCs were matured by adding 2 ng/ml IL-1β, 1000 U/ml IL-6 and 10 ng/ml TNFα (all from R&D Systems, Minneapolis, USA) (1997). DCs were recovered after a further 48 hours.

Mononuclear cells from tonsils were kindly provided by P Murray and E Nagy. Tonsils were obtained after tonsillectomy for recurrent acute tonsillitis, minced and dissociated by mechanical means and mononuclear cells were collected by Ficoll separation.

## TCR gene transduction.

CBMCs were retrovirally transduced with a TCR, specific for the HLA A11-restricted SSCSSCPLSK (SSC) peptide of the LMP2 protein of Epstein Barr virus, as previously described (Frumento *et al*, 2013). Briefly, cells were transduced with retrovirus 48 hours after activation. The retrovirus used was the pMP71-PRE vector (provided by C. Baum, Hannover, Germany) with genes encoding TCR α and β chains isolated from an EBV-specific $CD8^+$ T cell clone that targets the HLA A*1101-restricted epitope SSC derived from the viral protein LMP2 (Zheng *et al*., 2015). To generate the retrovirus, Phoenix amphotropic packaging cells were transfected with the pMP71-PRE vector using FuGENE HD (Roche, Basel, Switzerland). After 48 hours the retroviral supernatant was recovered. Preactivated cells were seeded at 4–6 × 106 cells/well in 1 ml RPMI onto 6-well plates coated with retronectin (Takara, Shiga, Japan). Retroviral supernatant (1.5 ml/well) or medium alone (mock-transduced) was added to each well and centrifuged for 1 h × 800 g at 30°C. Also described are generation of DCs, their loading with the peptide, and re-stimulation of transduced T cells with peptide-pulsed DCs.

## Cell activation and treatment.

Cells were activated, or re-activated, with either of the following stimuli.

Anti-CD3: cells were incubated with 66 ng/ml anti-CD3 antibody (OKT3), plus 300 U/ml IL-2 (Miltenyi). Anti-CD3 and crosslinked anti-CD28: cells were incubated with 66 ng/ml OKT3 plus a mix of 66 ng/ml LEAF anti-CD28 (BioLegend, San Diego, CA, USA) and 66 ng/ml rat anti-mouse IgG1 (BioLegend), and 50 U/ml IL-2. CD3/CD28 beads: Dynabeads T Activator CD3/CD28 (Life Technologies, Grand Island, NY) beads were incubated with CBMCs at 1:1 ratio in the presence of 30 U/ml IL-2. PHA: cells were incubated with 1% PHA M (Life Technologies, Carlsbad, CA, USA), plus 50 U/ml IL-2. SEB: cells were incubated with 1 μg/ml SEB (Sigma-Aldrich, St. Louis, MO, USA), plus 50 U/ml IL-2. From day 2, IL-2 100 U/ml, for stimulation with soluble anti-CD3, or 30 U/ml, for the other cases, was added thrice a week. Phenotype was checked thrice a week. When the percentage of $CCR7^+$/$CD45RA^+$ $CD8^+$ T cells dropped below 20%, half of the medium was replaced with fresh medium containing either IL-2, IL-4, IL-6, IL-7, IL-15 or IL-21 (all from Miltenyi), or combinations thereof, at final concentrations of 30 U/ml, 25 ng/ml,

10 ng/ml, 25 ng/ml, 50 ng/ml, and 50 ng/ml, respectively. Thrice a week, half of the culture medium was replaced with new medium plus cytokine(s). Re-activation by phorbol myristate acetate (PMA) plus ionomycin (Cell Stimulation Cocktail, eBioscience, San Diego, CA, USA) was performed exactly following manufacturer's instructions. BMS 493 (Tocris, Brisol, UK) was preincubated 3 hours before addition of IL_7 and/or all-trans-retinoic acid (ATRA, Sigma Aldrich).

Flow cytometry.

The antibodies used for cell membrane staining are listed in Table S5. Gating strategy involved selection of single cells and use of a "dump channel" including either 7-aminoactinomycin D (BD, San Jose, CA, USA) and PerCP-conjugated anti CD14, CD16 and CD19, or Live/Dead Fixable Violet (Thermo Fisher Scientific, Wilminghton, DE, USA) and Pacific Blue-conjugated anti CD14, CD16 and CD19. Proliferation was evaluated by staining cells with 1 μM CFSE. For intracellular staining, cells were fixed and permeabilized using the FIX&PERM kit (ADG, Kaumberg, Austria) or Fixation/Permeabilization Solution Kit (BD) for staining of cytokines or TFs, respectively. Indirect staining was carried out for E2F-1 (clone 8G9, Novus Biologicals, Littleton, CO, USA) and KAT2B (clone EPR2670, Abcam, Cambridge, MA, USA). In this case a PE- conjugated rat anti-mouse IgG1 (clone RMG1-1, BioLegend, San Diego, CA, USA) and an APC-conjugated goat ant-rabbit IgG (R&D, Minneapolis, MN, USA) were used, respectively. In some experiments cells were enumerated using Trucount Beads (BD). Transduced lymphocytes were identified using HLA A*1101:SSC peptide-specific pentamers and Tag/PE (Proimmune, Oxford, UK). Flow cytometry was performed on either a FACSCanto II, an LSR II, or a Fortressa (BD) flow cytometer. Quantifications were made on the basis of FACSDiva and FlowJo (both from BD) softwares.

Cytotoxicity assay.

Cytotoxicity of transduced T cells was assessed in a standard $^{51}$Cr assay. Briefly, HLA A*1101-transduced T2 cells were loaded with the concentrations of SSC peptide indicated in the text, then used as targets at 2500 cells/well in a 5 hrs test.

ATAC-seq library preparation and analysis

For sequencing the open chromatin regions in the CD8$^+$ T cell subsets, we used a previously published method with slight modification (Brignall *et al*, 2017). In brief, 50,000 cells were sorted on a cell sorter (FACSAria, BD) and pelleted by centrifugation at 500 × *g* and 4°C for 10 min. The cell pellet was washed once with cold PBS and centrifuged again. Pellets were resuspended in a 50-µl reaction cocktail containing 1 µl of 0.5% Digitonin (Promega, Madison, WI, USA), 2.5 µl of Tn5 transposase and 25 µl of TD buffer (Nextera DNA library preparation kit, Illumina, San Diego, CA, USA) and incubated for 30 min at 37°C with gentle shaking. The tagmented DNA was then purified using Minielute PCR Purification kit (Qiagen, Hilden, Germany) and eluted into 11 µl of elution buffer. The transposed DNA was then amplified using 2.5 µl of indexing primers and 25 µl of Nextera PCR master mix according to the PCR protocol previously described (Buenrostro *et al*, 2013). PCR clean-up was then performed using AMPure XP beads (Beckman Coulter, Brea, CA, USA), and resuspended in 30 µl of resuspension buffer. The libraries were quantified using Qubit fluorometer (Life Technologies), and the size was detemined using TapeStation (Agilent, Santa Clara, CA, USA). Sequencing was performed on a High Throughput Benchtop NextSeq 500/550 Sequencer using NextSeq® 500/550 High Output Kit v2 - 75 cycles (Illumina, San Diego, CA, USA).

ATAC-seq data was aligned to the human genome (hg19) using Bowtie2 v2.2.9 with the settings: –very-sensitive-local (Langmead and Salzberg, 2012). Peaks were called on the alignments using MACS2 v2.2 with settings: --keep-dup=auto –nomodel –shift -100 –extsize 200 (Zang *et al*, 2008). Peaks were annotated with closest gene (by RefSeq transcription start site) and average tag counts (400bp region centred on peak summit) using annotatePeaks from HOMER v4.8. Motif enrichment at peak regions was assessed using the HOMER program findMotifsGenome (Heinz *et al*, 2010). DACs were identified by pairwise comparisons in the R package DESeq2 (Love *et al*, 2014). Genome-wide average profile data at TSS and enhancer sites was obtained using ngs.plot v2.63 (Shen *et al*, 2014). Heatmaps of DACs and mapped read profiles at selected genomic regions were obtained using fluff v2.1.4 (Georgiou *et al*,

2016). ATAC-seq data have been deposited in Gene Expression Omnibus, accession number GSE120618, public release December 2018.

## Microarray analysis.

Gene expression analysis was performed on 3 CD8$^+$ $T_{Nrev}$ at day 19 and 3 CD8$^+$ $T_N$ samples. RNA extraction was performed using RNeasy columns (Qiagen). Source RNA was confirmed as high quality by use of a Bioanalyzer 2100 (Agilent). RNA Integrity Numbers of 6.0 were confirmed for all samples using a RNA 6000 Pico Chip kit (Agilent). 25ng of each source sample RNA was labeled with Cy3 dye using the Low Input Quick Amp Labelling Kit (Agilent). A specific activity of greater than 6.0 was confirmed by measurement with a spectrophotometer. 600 ng of labeled RNA was hybridized to SurePrint G3 Human 8x60K microarray slides (Agilent). After hybridization, slides were scanned with a High Resolution C Scanner (Agilent), using a scan resolution of 3 mm. Feature extraction was performed using Feature Extraction Software (Agilent), with no background subtraction. Extracted data were normalized using the R 3.4 software environment with the limma 3.32.5 analysis package (Smyth, 2004). Log transformed expression values were analyzed in limma using a moderated paired t-test. Microarray data that support the findings of this study have been deposited in Gene Expression Omnibus, accession number GSE114812, public release December 2018.

## RNA-seq analysis

RNA extraction was performed using RNeasy columns (Qiagen). TruSeq mRNA library preparation and sequencing was carried out by Edinburgh Genomics, The University of Edinburgh. Sequencing data were generated using NovaSeq 50PE.

## Statistics

Quantifications of events from flow cytometry were made using FACSDiva and FlowJo (both from BD) softwares.

Genes were classified as differentially expressed if Benjamini-Hochberg adjusted p-value $<0.05$ and absolute fold change $>2$. Peaks discovered from the ATAC-seq analysis were classified as differentially

accessible in a given pairwise comparison if **Benjamini-Hochberg adjusted p-value <0.1 and absolute log2 fold change >1**. Pathway enrichment analysis was performed using G:profiler (Reimand *et al*, 2016) with a threshold adjusted p-value of <0.05 for reporting significant enrichments. Gene-set enrichment analysis (GSEA) was performed using the Bioconductor R package GAGE (Luo *et al*, 2009). The Hallmark and Canonical Pathways gene sets were downloaded from Molecular Signatures Database (MSigDB) (Liberzon *et al*, 2015). GSEA was also used to assess enrichment of published gene signatures for $T_{SCM}$ up/down-regulated vs $T_N$ (Gattinoni *et al*, 2011) and $T_{MNP}$ up/down-regulated vs $T_N$ (Pulko *et al*, 2016). $T_{MNP}$ up/down-regulated genes vs $T_N$ were extracted from published RNAseq count data downloaded from NCBI's Gene Expression Omnibus (GSE80306). Differentially expressed genes (DEGs) with Benjamini-Hochberg adjusted p-value<0.05 and absolute fold change >2 were identified using the R package DEseq2. Genomic Regions Enrichment of Annotations Tools (GREAT) (McLean *et al*, 2010) was used to identify functional enrichments within genes annotated as closest to differentially accessible chromatin sites. Functional and network analysis was performed on the pool of significant genes using Ingenuity Pathway Analysis (Qiagen).

In all the other cases statistical analysis was carried out using Graph Pad Prism software or Excel 2016. Results in graphs are reported as mean ± 1SD.

## SUPPLEMENTAL REFERENCES


Brignall R, Cauchy P, Bevington SL, Gorman B, Pisco AO, Bagnall J, Boddington C, Rowe W, England H, Rich K et al (2017). Integration of kinase and calcium signaling at the level of chromatin underlies inducible gene activation in T cells. *J. Immunol*. 199, 2652-2667.

Buenrostro JD, Giresi PG, Zaba LC, Chang HY, Greenleaf WJ (2013). Transposition of native chromatin for fast and sensitive epigenomic profiling of open chromatin, DNA-binding proteins and nucleosome position. *Nat. Methods* 10, 1213-1218.

Georgiou G, van Heeringen SJ (2016). fluff: exploratory analysis and visualization of high-throughput sequencing data. *PeerJ* 19, e2209.

Heinz S, Benner C, Spann N, Bertolino E, Lin YC, Laslo P, Cheng JX, Murre C, Singh H, Glass CK (2010). Simple combinations of lineage-determining transcription factors prime cis-regulatory elements required for macrophage and B cell identities. *Mol. Cell* 38, 576-589.

Langmead B, Salzberg S (2012). Fast gapped-read alignment with Bowtie 2. *Nat. Methods* 9, 357-359.

Liberzon A, Birger C, Thorvaldsdóttir H, Ghandi M, Mesiro, JP, Tamayo P (2015). The Molecular Signatures Database (MSigDB) hallmark gene set collection. *Cell Syst*. 1, 417-425.

Love MI, Huber W, Anders S (2014). Moderated estimation of fold change and dispersion for RNA-seq data with DESeq2. Genome Biol. 15, 550.

Luo W, Friedman MS, Shedden K, Hankenson KD, Woolf PJ (2009). GAGE: generally applicable gene set enrichment for pathway analysis. *BMC Bioinformatics* 10, 161.

McLean CY, Bristor D, Hiller M, Clarke SL, Schaar BT, Lowe CB, Wenger AM, Bejerano G (2010). GREAT improves functional interpretation of cis-regulatory regions. *Nat. Biotechnol*. 28, 495-501.

Reimand J, Arak T, Adler P, Kolberg L, Reisberg S, Peterson H, Vilo J (2016). g:Profiler-a web server for functional interpretation of gene lists. *Nucleic Acids Res*. 44, W83-89.

Shen L, Shao N, Liu X, Nestler E (2014). ngs.plot: Quick mining and visualization of next-generation sequencing data by integrating genomic databases. *BMC Genomics* 15, 284.

Zhang Y, Liu T, Meyer CA, Eeckhoute J, Johnson DS, Bernstein BE, Nusbaum C, Myers RM, Brown M, Li W et al (2008). Model-based analysis of ChIP-Seq (MACS). *Genome Biol*. 9, R137.

Zheng Y, Parsonage G, Zhuang X, Machado LR, James CH, Salman A, Searle PF, Hui EP, Chan AT, Lee SP (2015). Human Leukocyte Antigen (HLA) A*1101-Restricted Epstein-Barr Virus-Specific T-cell Receptor Gene Transfer to Target Nasopharyngeal Carcinoma. *Cancer Immunol. Res*. 3, 1138-1147.
